# Comprehensive genome-wide transcription factor analysis reveals that a combination of high affinity and low affinity DNA binding is needed for human gene regulation

**DOI:** 10.1186/1471-2164-16-S7-S12

**Published:** 2015-06-11

**Authors:** Junbai Wang, Agnieszka Malecka, Gunhild Trøen, Jan Delabie

**Affiliations:** 1 Pathology Department, Oslo University Hospital - Norwegian Radium Hospital, Montebello 0310, Oslo, Norway

## Abstract

**Background:**

High-throughput *in vivo *protein-DNA interaction experiments are currently widely used in gene regulation studies. Hitherto, comprehensive data analysis remains a challenge and for that reason most computational methods only consider the top few hundred or thousand strongest protein binding sites whereas weak protein binding sites are completely ignored.

**Results:**

A new biophysical model of protein-DNA interactions, BayesPI2+, was developed to address the above-mentioned challenges. BayesPI2+ can be run in either a serial computation model or a parallel ensemble learning framework. BayesPI2+ allowed us to analyze all binding sites of the transcription factors, including weak binding that cannot be analyzed by other models. It is evaluated in both synthetic and real *in vivo *protein-DNA binding experiments. Analysing ESR1 and SPIB in breast carcinoma and activated B cell-like diffuse large B-cell lymphoma cell lines, respectively, revealed that the concerted binding to high and low affinity sites correlates best with gene expression.

**Conclusions:**

BayesPI2+ allows us to analyze transcription factor binding on a larger scale than hitherto achieved. By this analysis, we were able to demonstrate that genes are regulated by concerted binding to high and low affinity binding sites. The program and output results are publicly available at: http://folk.uio.no/junbaiw/BayesPI2Plus.

## Background

High-throughput *in vivo *protein-DNA binding experiments such as ChIP-chip and ChIP-seq are currently widely used to study gene regulation. Identification of transcription factor (TF) binding sites is an essential step to understand TF function and gene regulatory networks [1]. In such analyses, raw reads of ChIP-seq experiments are mapped to a human reference genome. Subsequently a peak-calling program is used to detect putative TF binding sites. However, two problems arise by doing so. First, the identified TF binding sites are dependent of the threshold value used by the peak-calling program. If stringent criteria are applied, many potentially functional TF binding sites with weak binding (i.e. low binding affinities or low ChIP-seq tag count) may be eliminated [2]. If on the other hand non stringent cutoff values are chosen, many false binding sites are identified. Second, ChIP-seq experiment may not necessarily identify the direct TF-DNA interactions due to the inherent inability of current ChIP-seq or ChIP-chip technology to distinguish direct versus indirect protein-DNA interactions [3,4]. So far, the first problem has been ignored [5]. Several studies have addressed the second problem only partially. Vallania et al. [6] focused on identifying only functional direct TF binding sites by using a computational method based on weight matrices, comparative genomics, and gene expression profiles; Gordan et al [3] developed a method to separate direct TF binding from indirect TF binding in yeast ChIP-chip data but it has not been used to analyze human ChIP-seq experiments. In addition, the method requires that both *in vivo *binding data and *in vitro *DNA binding motifs are available; more recently, Bailey et al [7] proposed an interesting statistical method "Central Motif Enrichment Analysis" (CentriMo) to predict direct DNA binding sites from ChIP-seq data. Unfortunately, the program only works with equal-length genomic sequences and does not provide information about functional indirect TF binding.

In the present work, a more comprehensive computational approach, BayesPI2+, is developed for analyzing *in vivo *high-throughput protein-DNA interaction data, where the above-mentioned problems are solved. Especially, non stringent peak calling cutoff values can be used allowing the inclusion of many weak protein binding sites in the data analysis. The newly developed BayesPI2+ is a C program that is based on a biophysical model of protein-DNA interactions [8,9] and that can be run in both a serial computation model and in a parallel ensemble learning framework. BayesPI2+ estimates protein binding energy matrix (PBEM), protein concentration (or chemical potential) in a solution [8], and differential binding affinity (dbA) of protein-DNA interactions, through *in vivo *protein-DNA interaction experiments. Based on these predicted features, BayesPI2+ allowed to distinguish high and low affinity protein binding sites called here for reasons of simplicity, type I and type II TF binding [10]. The novel method was first tested in both synthetic ChIP-seq data and several real *in vivo *protein-DNA binding data sets. Then, gene regulatory difference between the predicted type I and type II TF binding sites was investigated in two human ChIP-seq experiments, the estrogen receptor α (ESR1) using the MCF7 breast cancer cell line and the SPIB TF using the HBL1 activated B cell-like diffuse large B-cell lymphoma (ABC DLBCL) cell line. ESR1 is a member of the nuclear receptor family of ligand-activated TFs and is involved in the development and progression of breast cancer [11]. SPIB belongs to ETS-family of TFs and is required for the survival of ABC DLBCL cells [12]. Putative SPIB type I and type II binding sites were verified by *in vitro *protein-DNA interaction experiments. Subsequently, we tested whether these binding sites have an effect on gene expression. Of interest, our analysis showed that the binding of TF to both type I and type II binding sites is important for gene expression.

## Results

### Distinguishing type I versus type II TF binding sites using synthetic ChIP-seq data sets

**Figure 1:**
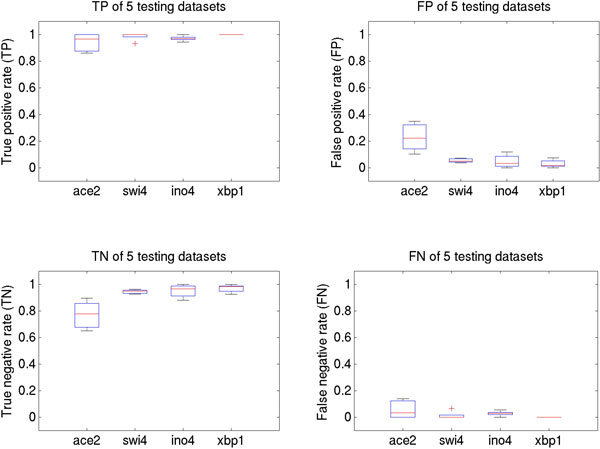
**Error rates of distinguishing type I versus type II TF binding sites in synthetic ChIP-seq data sets by using a serial version of BayesPI2+**. Error rates (i.e. TP - true positive rate, FP - false positive rate, TN - true negative rate, and FN - false negative rate) are displayed in the box plot, which were estimated by randomly splitting training and testing data multiple times. The inferred best TF PBEM from training data was used to compute *dbA *and to distinguish type I versus type II TF binding sites in the testing data.

Here, BayesPI2+ was first used to infer the PBEM and the chemical potential from a synthetic training data set [8]. Then the best PBEM, i.e. a PBEM with the highest motif similarity score to a known consensus sequence motif, was chosen to predict protein binding sites in a data test set. Results of a serial computation, testing error rates (i.e. true positive (TP), true negative (TN), false positive (FP) and false negative rates (FN)), are displayed in Figure 1. The rates were estimated by comparing to known direct TF binding sites (i.e. expected TF binding motif in the synthetic DNA sequence with a synthetic Z-score > 0.25) with the predicted direct TF binding sites (i.e. identified by applying fuzzy neural gas algorithm on differential binding affinity (dbA)). It takes around fifteen to thirty minutes for a serial version of BayesPI2+ to complete the prediction in one synthetic data set.

In Additional file 1: Figure S1, test error rates by using a parallel ensemble BayesPI2+ learning are shown. In this analysis, both meta-PBEM and meta-chemical-potential (mean of PBEMs from multiple predictions and the corresponding chemical potential) were computed by five times random splitting training and testing (i.e. 50%) data. These predictions were completed in five to eight minutes. The overall prediction accuracy with this method is almost the same as what was obtained by a serial computation. Of interest, the longer the binding motif, the better the test error rates. TP and TN of ACE2 with a binding motif of 6bp are between 70% and 90% whereas the TP and TN of the other TFs with binding motifs between 8bp and 12bp are approximately 95%. Thus, both serial and parallel ensemble BayesPI2+ learning are able to distinguish type I versus type II TF binding sites in synthetic ChIP-seq datasets.

### Distinguishing type I versus type II TF binding sites by using *in vivo *protein-DNA binding data

#### Prediction of PBEM in five data sets of various sizes

**Figure 2:**
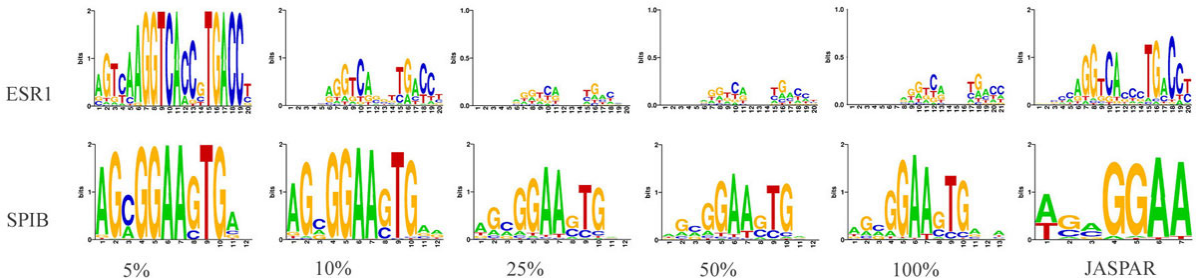
**Comparisons of predicted PBEMs based on various sizes of called peaks**. A parallel ensemble learning and a serial computation of BayesPI2+ were applied on a portion of randomly selected (i.e. 5%, 10%, 25%, 50%) and all (i.e. 100%) called peaks, respectively. Sequence log representations of the predicted best PBEMs (100% called peaks) and the meta-PBEMs (i.e. 10 times random selection of called peaks) from various input sizes are displayed in the figure. The last column is the known ESR1 and SPIB binding motifs from JASPAR database.

Encouraged by the results from synthetic ChIP-seq data, BayesPI2+ was applied to five human ChIP-seq data sets [12-14]: NRSF, ESR1, CTCF, SPIB and STAT1 with 6k, 17k, 27k, 43k and 74k called peaks, respectively. For each TF, a parallel ensemble version of BayesPI2+ was applied to randomly selected peaks constituting 5%, 10%, 25%, and 50% of called peaks, respectively. Each random selection was repeated ten times to estimate the meta-PBEM. A serial computation of BayesPI2+ was used to infer the best PBEM based on all called peaks, i.e. 100% of input data. Computational costs of all predictions were recorded in Additional file 1: Figure S2. As expected, a linear increase of CPU time was observed when the input size is increased from 5% to 100% of called peaks. Sequence logo representations of predicted meta-PBEMs based on multiple random selections of 5% to 50% of called peaks and the best PBEM from 100% called peaks for (ESR1, SPIB) and (NRSF, CTCF, STAT1) are shown in Figure 2 and Additional file 1: Figure S3, respectively. Two observations can be drawn from the analysis as illustrated by the figures: 1) the predicted PBEMs from serial computation are similar to the meta-PBEMs, but positional trends in information content are observed across data of different input size with the exception of data for NRSF that has the fewest called peaks; 2) the positional trends in information content are near maximum when the number of input peaks used for analysis is greater than and equal to 25% of called peaks. By considering both computational efficiency and the prediction accuracy, the present results indicate that multiple random selections of ~25% of called peaks are sufficient for a parallel ensemble version of BayesPI2+. Especially, if the input data size is large (i.e. > 10000 called peaks) then a parallel ensemble learning of BayesPI2+ will be preferred in the data analysis.

#### Distinguishing type I versus type II TF binding sites in human ChIP-seq data sets

**Figure 3:**
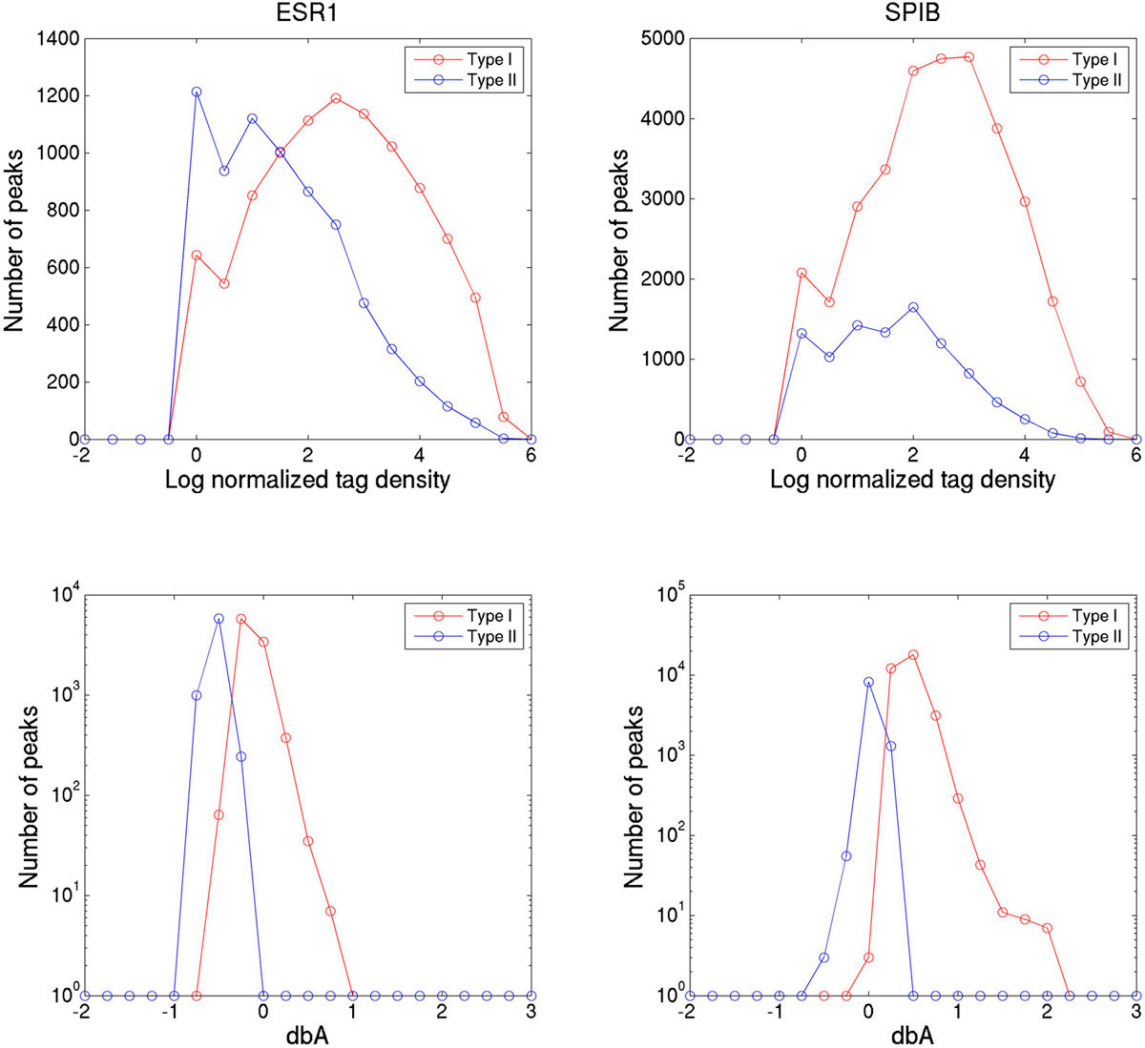
**Distribution of ChIP-seq tag density and differential binding affinity (*dbA*) for predicted type I and type II TF binding sites**. By random drawing 25% of all called ChIP-seq peaks ten times, meta-PBEMs of human ESR1 and SPIB were estimated, respectively, by a parallel ensemble version of BayesPI2+. Expected P-values, *dbA*, and classification of type I and type II TF binding for all called peaks were based on these meta-PBEMs. In the figure, left and right panels illustrate distributions of log normalized ChIP-seq tag density and *dbA *of predicted type I (red lines) and type II TF binding sites (blue lines) for ESR1 and SPIB, respectively.

Subsequently, distinguishing type I versus type II TF binding sites was analyzed in five human ChIP-seq datasets. First, the meta-PBEM and meta-chemical-potential of each TF, as estimated by random selection of 25% of called peaks ten times (Additional file 1: Figure S4), were used to compute the expected P-values and dbA for every binding site. Then, a fuzzy neural gas algorithm was applied to dbA to classify called peaks into type I and type II TF binding sites, where the expected P-value of type I and type II TF binding is <0.09 and > = 0.09, respectively. Finally, the significance of meta-PBEM for identification of TF binding sites sequences was evaluated by fitting the predicted TF binding affinities on DNA sequences and the measured ChIP-seq tag density to a linear regression model, then transforming the regression coefficients to T-values [15], which measures the dependence between the estimated meta-PBEM and the observed ChIP-seq tag counts. The results show that the meta-PBEMs of 5 tested human TFs are significantly correlated with the predicted type I (direct) TF binding site sequences for 5 TFs, respectively (Additional file 1: Figure S5). Distributions of both tag densities and dbA are shown in Figure 3 and Additional file 1: Figure S6 for the 5 TFs, respectively. Though there is a large overlap of ChIP-Seq tag densities between the type I and type II TF binding, the distribution of dbA levels clearly separates the type I from the type II TF binding, except for few sites with almost zero dbA. Therefore, the estimated dbA level is a good parameter to distinguish two types of TF binding sites, whereas the raw tag counts of called peaks are not suited to accomplish this.

#### Distinguishing type I versus type II TF binding sites in yeast ChIP-chip data sets

ChIP-seq is a high resolution experiment that identifies putative protein binding site sequences of short and equal-length, approximately 200 bp. We therefore also tested whether BayesPI2+ can predict type I TF binding sites by using unequal-length genomic sequences. Here, a series computation of BayesPI2+ was applied to four yeast ChIP-chip experiments with TFs ACE2, SWI4, INO4, and XBP1 in rich medium conditions [16]. The putative protein binding sites are positioned on ~6725 yeast intergenic regions of unequal-length varying between 50 bp to ~ 2700 bp, with a median length of ~360 bp. The results indicate that only three of four yeast TFs (ACE2, SWI4 and INO4) obtained good PBEM by using BayesPI2+ (i.e. the motif similarity scores [8,17] between the best PBEM and the SGD consensus sequences was >0.8; Additional file 1: Figure S7). A plot of P-values, reflecting the confidence level of detecting a binding site, [16] against the number of binding sites with p values below the defined p-value can be made for the four yeast ChIP-chip experiments (Additional file 1: Figure S8). The plot shows that there are more than 100 binding sites with confidence level P < 0.005 for ACE2, SWI4 and INO4, respectively. However, none of the XPB1 binding targets passed the same level of significance of binding. The poor binding prediction for XBP1 can be explained either by true weak protein binding or poor quality of the ChIP-chip experimental data. Therefore, only ACE2, SWI4 and INO4 were considered in the subsequent data analysis.

**Figure 4:**
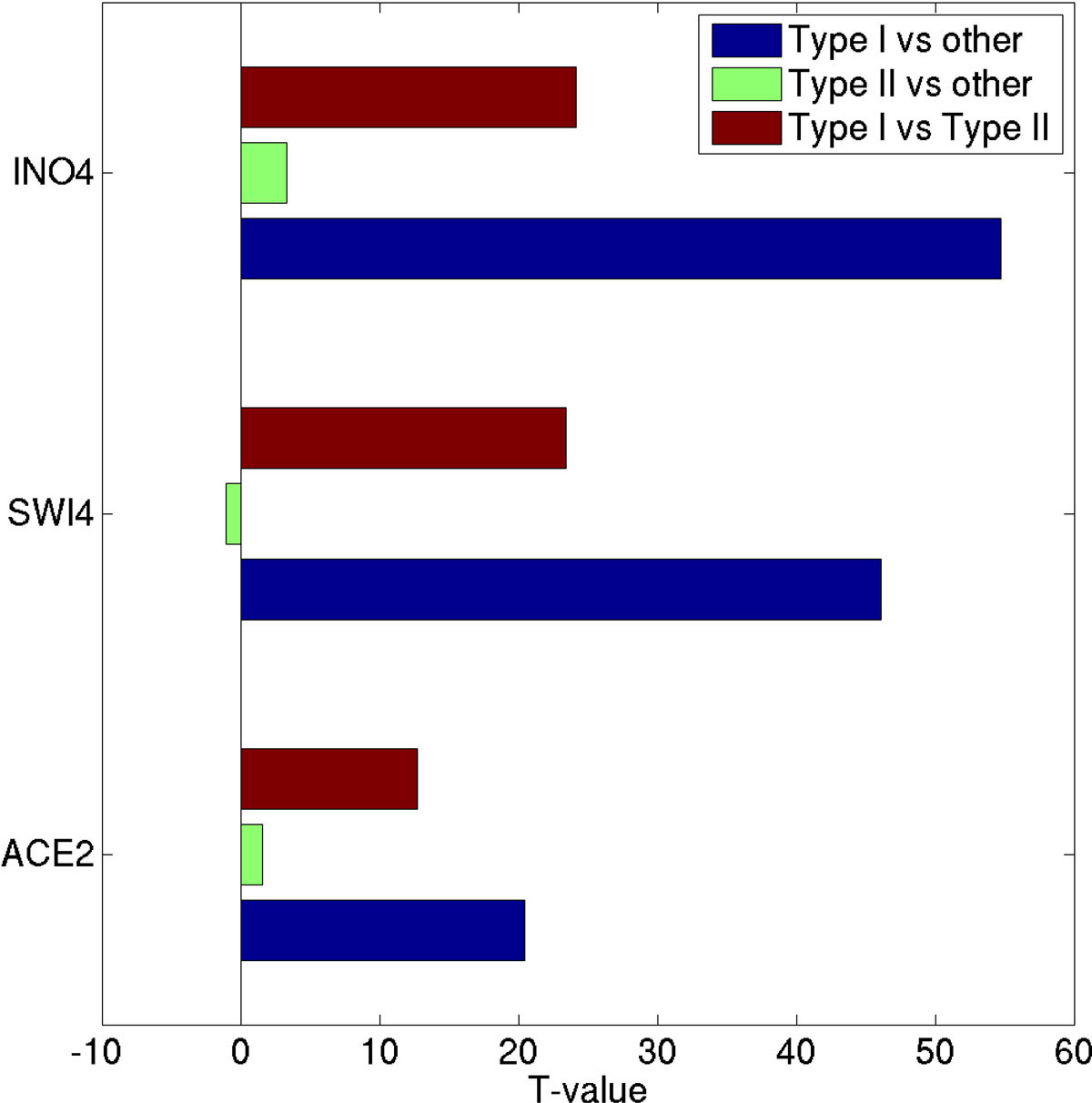
**PBEM enrichment tests in type I and type II TF binding sites for three yeast ChIP-chip experiments**. By using a serial version of BayesPI2+, the best representative PBEMs were inferred from yeast ChIP-chip experiments for ACE2, SWI4 and INO4, respectively. Expected P-values, dbA, and classification of type I and type II TF binding sites (unequal-length yeast intergenic region) were based on these predicted PBEMs. In the figure, enrichment of PBEMs in predicted type I, type II TF binding sites, and the rest of intergenic regions ('Other') are shown by bar plots, where T-values were obtained by a two-tailed t-test (i.e. TF binding affinity in type I versus that in type II TF binding, in type II versus that in "Other", and in type I versus that in type II TF binding).

Yeast intergenic regions with either low or high affinity binding sites (i.e. binding confidence level P <0.1) were considered as containing putative TF binding sites. Thus, about 381, 419, and 675 yeast intergenic regions with binding sites for ACE2, SWI4 and INO4, respectively, were identified. The inferred PBEM from a serial computation analysis was used to calculate dbA and the expected P-value. In this analysis, each selected intergenic region was randomly shuffled 5000 times. Then, type I and type II TF binding targets were classified by applying the fuzzy neural gas algorithm on the calculated dbA. Interestingly, for the three yeast TFs, no gene seems to be regulated by both type I and type II TF binding sites, where the assignment of TF binding sites to putative target genes was based on a published annotation [16]. To verify the predicted type I TF binding sites, the best predicted PBEM of each TF was used to compute *in silico *TF binding affinities on DNA sequences for type I, type II TF binding sites, and the rest of intergenic regions, respectively. A two tailed t-test was then performed to compare the TF binding affinities between type I and type II TF binding sites, and between type I/II TF binding sites and the rest of intergenic regions, respectively. A bar plot of the T-values of the t-tests is displayed in Figure 4. The predicted best PBEM of each TF is highly enriched in the type I TF binding but no enrichment was found in type II TF binding. Similar results were obtained by using 234 collected yeast consensus sequence motifs (Additional file 1: Figures S9 and S10) [18]. Hence, the classification of type I and type II TF binding sites by using the dbA proves also robust when used to analyze unequal-length genomic sequences.

#### Comparison of predicted type I TF binding sites identified by BayesPI2+ and CentriMo in mouse ChIP-seq data sets

First, position specific weight matrices (PSWM) of three mouse TFs (MYC, STAT3, and OCT4) were obtained from JASPAR database. Based on the known PSWM of each mouse TF, type I (direct) TF binding sites were predicted by applying BayesPI2+ and CentriMo [7] on all called peaks (i.e. 500 bp genomic sequence centered on each peak) of three mouse ChIP-seq data sets, respectively. Here, default parameter settings were used in both programs. Results suggest that around 83%, 88%, and 92% of CentriMo predicted type I MYC, STAT3, and OCT4 binding sites are recovered by BayesPI2+, respectively. Thus, for predicting type I (direct) TF binding sites in *in vivo *protein-DNA interaction experiment, there is a good agreement between the new biophysical model BayesPI2+ and the published statistical method CentriMo.

### Differences in expression of genes with type I and type II TF binding sites for ESR1 and SPIB transcription factors

#### Functional ESR1 target genes are regulated by both type I and type II TF binding sites

**Figure 5:**
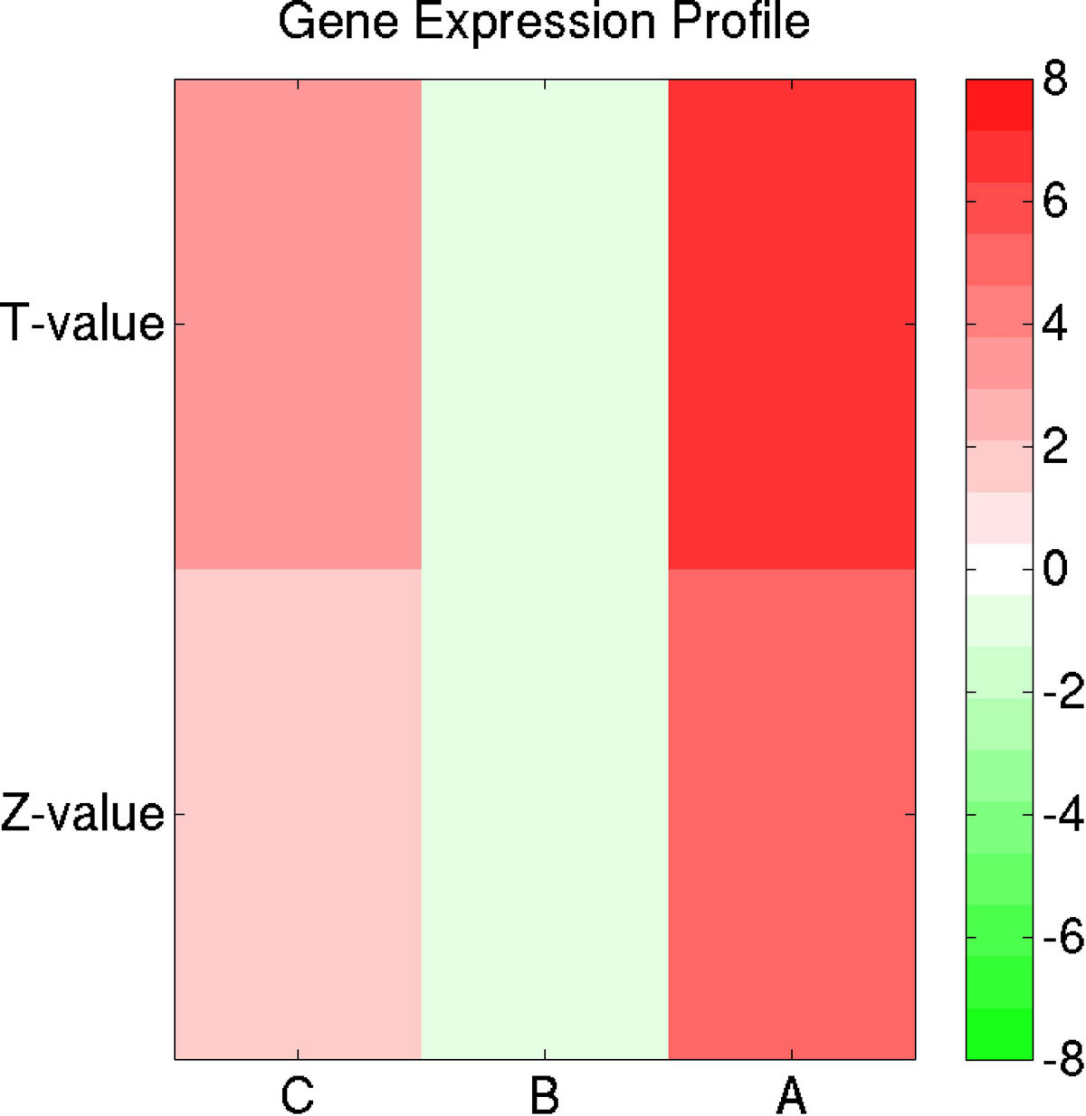
**Differential gene expression activities at three types of putative ESR1 target genes**. In the figure, T-value and Z-value are results of t-test and Mann-Whitney U test for gene expression profiles between putative ESR1 target genes (i.e. regulated by ESR1 'C', 'B', or 'A' genes) and the rest of genes in E2 treated MCF-7 breast cancer cell lines, respectively. Positive and negative T-values (Z-values) are colored by red and green, respectively.

After separating TF binding sites into type I and type II binding sites, we investigated the gene regulatory differences between the two. A functional study of putative ESR1 target genes, regulated by either type I or type II TF binding sites, was carried out in breast cancer cell line MCF-7 by using histone modification data [19], microarray gene expression profiles [14], and nucleosome density [20]. GREAT tool was used to find putative target genes regulated through either type I or type II TF binding sites. By this analysis, 6831 and 4321 putative target genes were identified for 9657 ESR1 type I TF binding sites and 7064 for ESR1 type II TF binding sites, respectively. Of interest, 3180 genes were regulated by both type I and type II TF binding sites, henceforth called 'A' genes; 3636 genes were regulated by type I TF binding sites only, called 'B' genes; and 1130 genes were exclusively regulated by type II TF binding sites, called 'C' genes in this manuscript. For the above-mentioned three classes of regulated genes, an enrichment test (i.e. t-test and Mann-Whitney U test) of gene expression activities (i.e. in E2 treated MCF-7 cells) between one class of genes and the rest of genes was performed. A heat-map of T-values and Z-values is shown in Figure 5. 'A' genes targeted by ESR1 are more highly expressed than 'B' and 'C' genes. Of interest and counter intuitively, 'C' genes have a higher expression level than 'B' genes. Distributions of ChIP-seq tag densities across the three classes of target genes are shown in Additional file 1: Figure S11. 'A' genes show the highest tag counts, as expected, 'C' genes show the lowest tag counts, and 'B' genes reveal intermediate tag counts. Taken together, our results suggest that ESR1 predominantly regulates 'A' genes with both type I and type II ESR1 binding sites. The functional consequence of exclusive type I or type II TF binding on gene expression seems to be less important.

**Figure 6:**
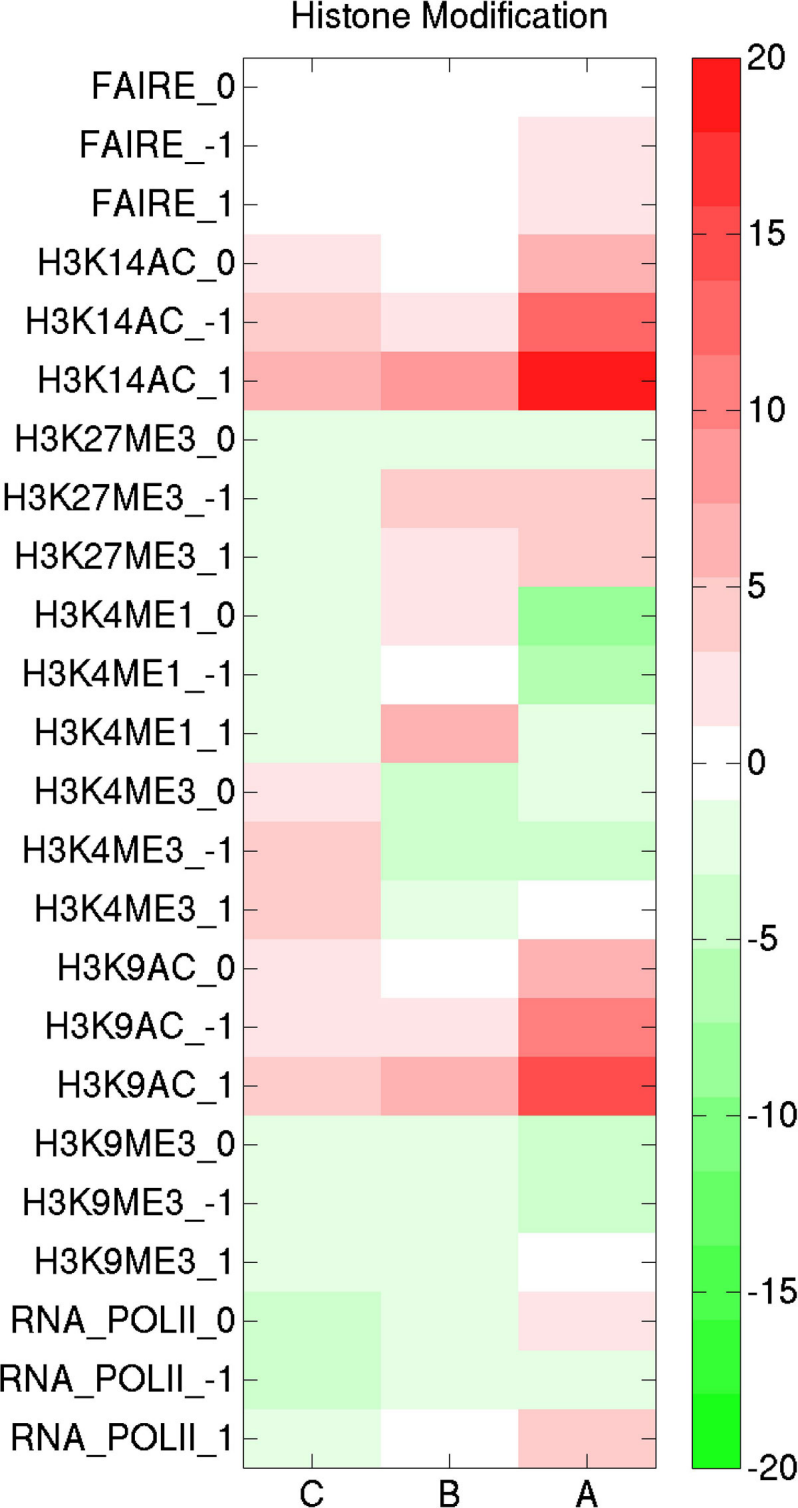
**Differential histone modifications at three types of putative ESR1 target genes**. A two-tailed t-test was used to evaluate significance of various marks between ESR1 target genes (i.e. 'C', 'B', and 'A' genes) and the rest of genes in genome. Positive and negative T-values are colored by red and green in the figure, where 0, +1 and −1 represent histone modifications in E2 treated MCF-7 breast cancer cell lines at gene body, 5 kb upstream and 5 kb downstream, respectively.

To further study the functional consequence of the different types of ESR1 binding to genes, histone modifications 5kb upstream, and 5 kb downstream of the genes were studied, respectively. Tag densities in E2-treated cells were normalized against control cells. A heat map of histone modifications upon E2-treatment is shown in Figure 6. T-values were obtained by performing the t-test for genes that were grouped in the three classes of ESR1 binding genes (i.e. 'A', 'B' and 'C') against the rest of genes, respectively. Of interest, the results are consistent with the analysis of gene expression analysis by microarray: 1) histone modification patterns are different among the three classes of ESR1 target genes; 2) only 'A' genes show decreasing nucleosome densities as evidenced by enriched FAIRE levels. Accordingly, RNA polymerase II levels are increased as well as histone acetylation (i.e. H3K14ac, H3K9ac). These observations are consistent with functional ESR1-DNA interactions predominantly through combined type I and type II binding of the transcription factor.

#### Robustness of the predicted ESR1 type I and type II TF binding site analysis

To test the robustness of the binding site prediction, a repeated analysis of gene expression profiles, ChIP-seq tag density, and histone modifications for the three classes of ESR1 target genes was performed, but this time with removal of the binding sites with low affinity. ESR1 binding sites of which both ChIP-seq tag count was less than 1.5 fold of the minimum tag count for type II TF binding sites and of which the dbA level was smaller than the maximum dbA in type II TF binding sites, were considered low affinity binding sites and removed. After filtering, 9355 and 2786 ESR1 type I and type II TF binding sites were remaining, respectively. Compared to the results before changing the thresholds, few ESR1 type I TF binding sites (~300) but more than half of the type II TF binding sites were lost. GREAT was then also used to find putative target genes for the new set of ESR1 binding sites. This time, 922 'C' genes, 3552 'B' genes, and 2450 'A' genes were found. Gene expression profile analysis, ChIP-seq tag density enrichment, and histone modifications analysis for these classes of genes are given in Additional file 1: Figures S12, S13, and S14, respectively. All results are consistent with those for the previous analyses, with the exception of a similar tag density for 'B' and 'C' genes after removal of low binding affinity sites. Functional annotation of the new three classes of ESR1 target genes by DAVID tool revealed that 'A' genes are highly enriched in pathways active in cancer, including the MAPK signaling pathway (Additional file 1: Table S1a and S1b).

#### Functional SPIB target genes are regulated by both type I and type II TF binding sites

**Figure 7:**
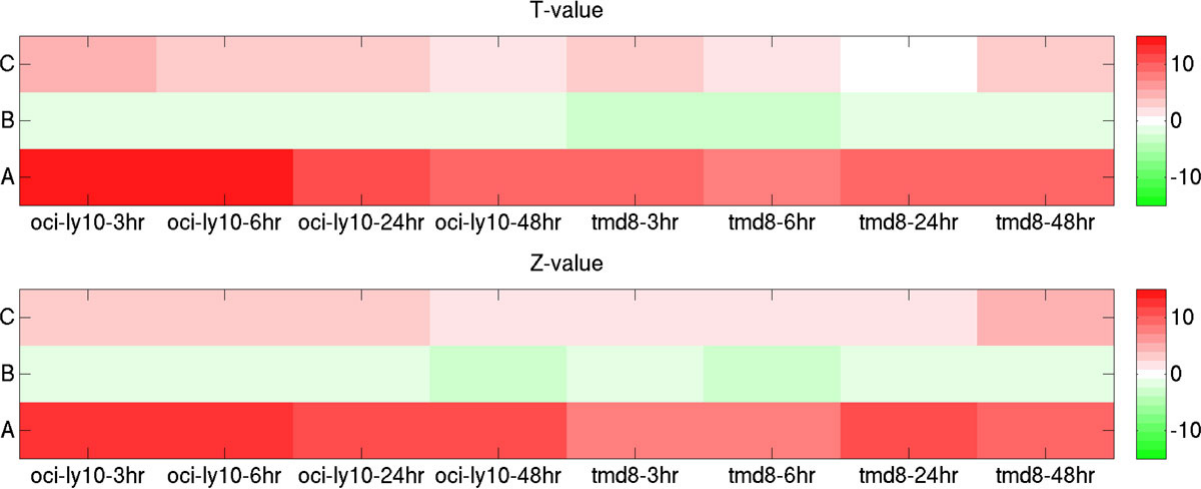
**Differential gene expression activities at three types of putative SPIB target genes**. In the figure, T-value and Z-value are results of t-test and Mann-Whitney U test, respectively, for gene expression profiles between putative SPIB target genes (i.e. 'C', 'B', and 'A' genes) and the rest of genes in lenalidomide treated ABC DLBCL cell lines (i.e. OCI-Ly 10, and TMD8; hr represents hours of treatment). Positive and negative T-values (Z-values) are colored by red and green in the figure.

**Figure 8:**
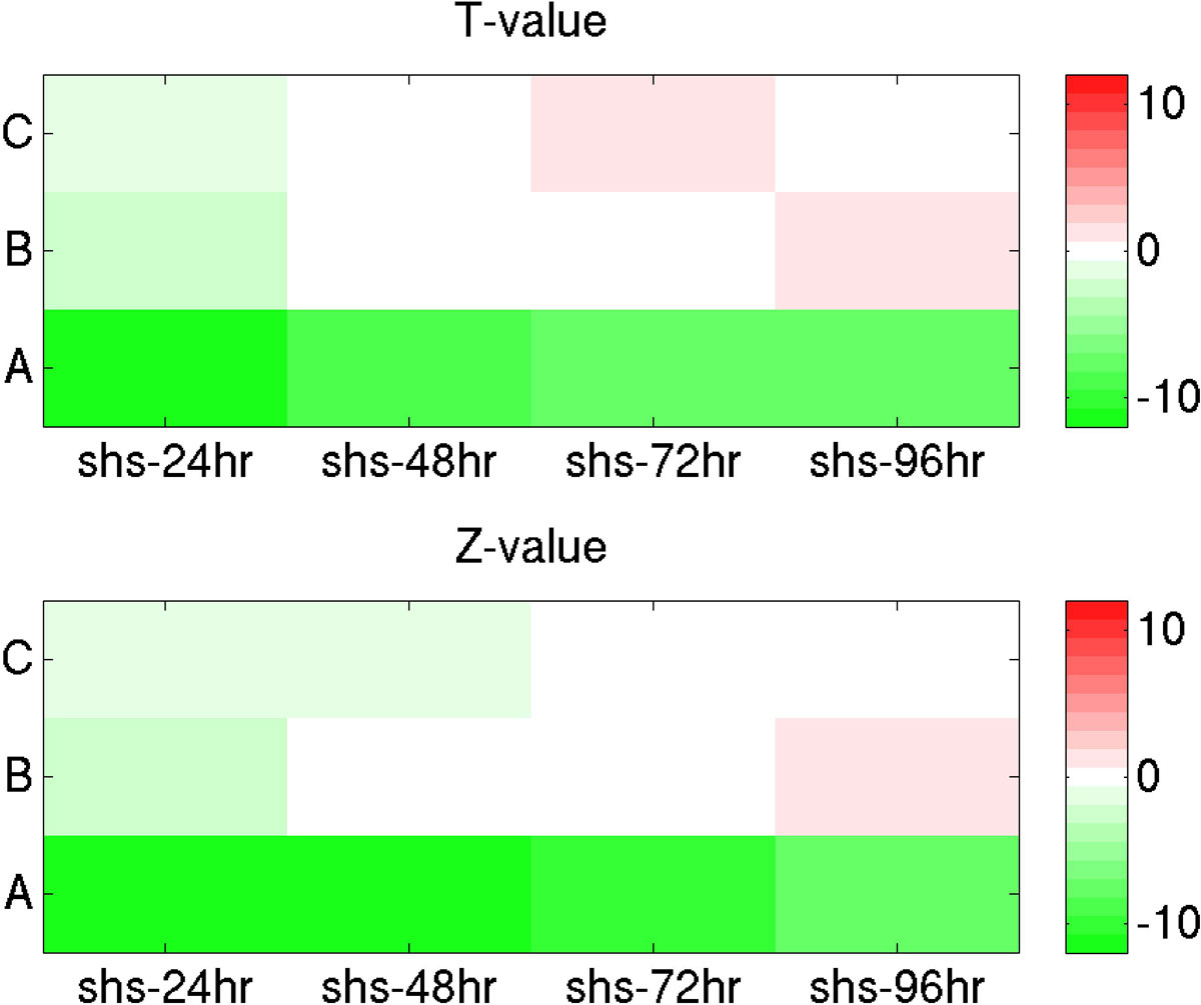
**Differential gene responses at three types of putative SPIB target genes after silencing of SPIB by RNA interference in ABC DLBCL cell lines**. In the figure, T-value and Z-value are results of t-test and Mann-Whitney U test for gene expression profiles between putative SPIB target genes (i.e. 'C', 'B', and 'A' genes) and the rest of genes, respectively, after silencing of SPIB by RNA interference in ABC DLBCL cell lines (i.e. HBL1; shs-hr represents silencing of SPIB in hours). Positive and negative T-values (Z-values) are colored by red and green in the figure.

Called SPIB ChIP-seq peaks [12] were first classified into type I (33561 peaks) and type II (9575 peaks) TF binding sites based on their estimated dbA levels from the meta-PBEM and meta-chemical-potential. Then, GREAT tool was used to identify 675, 6344 and 6911 genes that were possibly regulated by type II binding only ('C' genes), type I binding only ('B' genes) and by both ('A' genes), respectively. Subsequently, microarray gene expression analysis of the groups of genes in lenalidomide treated ABC DLBCL cell lines (i.e. OCI-Ly10 and TMD8) [12] was performed. T-test and Mann-Whitney U tests were used to evaluate the significance of differential expression between SPIB target genes (i.e. 'C', 'B', or 'A' genes) and the rest of genes in genome (Figure 7). Results reveal that SPIB target 'A' genes have the highest expression and that 'B' and 'C' genes only show a slight increase or decrease of the gene expression level. Additionally, silencing of SPIB by RNA interference in ABC DLBCL cell line HBL1 [12] results in significant repression of 'A' genes, but it does not have a strong impact on 'B' and 'C' genes as illustrated in Figure 8. These results strongly suggest that combined type I and type II transcription factor binding is necessary for gene activation. Functional annotation of the three groups of SPIB target genes by DAVID tool (Additional file 1: Table S2a) shows that 'A' genes are enriched in the MAPK signaling pathway (145 genes), in cancer in general (173 genes), in the T cell receptor signaling (67 genes), and B cell receptor signaling pathways (49 genes). Of interest, the latter pathways were not included among 'B' and 'C' genes. Thus, in analogy with what was demonstrated for ESR1 in breast cancer, a combination of SPIB type I and type II TF binding sites is needed to regulate gene expression in ABC DLBCL cells. SPIB is known to be up-regulated in ABC DLBCL [12]. Our data show that SPIB likely contributes to the characteristic up-regulation of B cell receptor pathway genes in ABC DLBCL.

#### Robustness of the predicted SPIB type I and type II TF binding site analysis

This analysis was performed following the same filtering criteria as for removing low affinity ESR1 binding sites. After filtering, 29378 and 4020 SPIB binding sites were remaining for type I and type II TF binding, respectively. 'C' genes are increased almost two-fold (1453 genes), 'A' genes were slightly reduced (6740 genes remaining), and 'B' genes were decreased by about one-forth (4742 genes remaining). Further analysis of lenalidomide treated ABC DLBCL microarray gene expression data and SPIB knockdown experiments in ABC DLBCL cells show that 'A' genes have the most significant response. These genes but not 'B' or 'C' genes, showed increased expression in lenalidomide-treated cells and decreased expression in knockdown experiments (Additional file 1: Figures S15 and S16). Of interest, 'A' genes were highly enriched in the same pathways (Additional file 1: Table S2b) as identified in the previous section.

### The nature of type II ESR1 and SPIB binding

**Figure 9:**
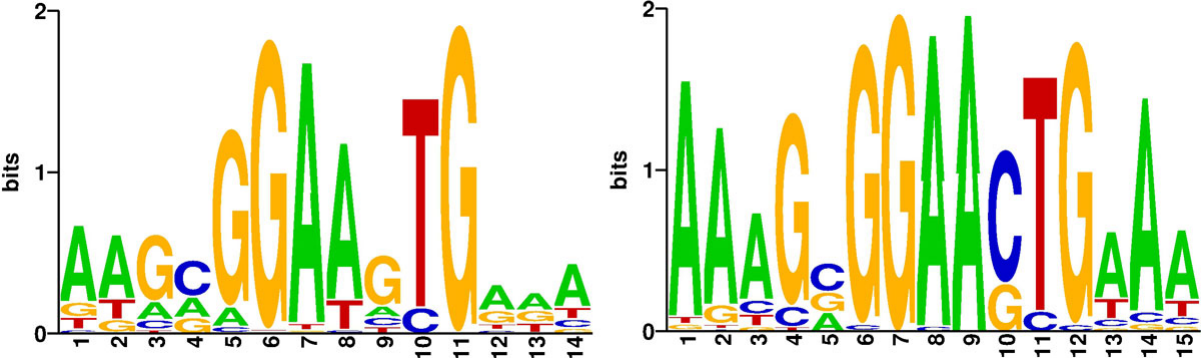
**Sequence log representation of the predicted primary and alternative SPIB PBEMs in ABC DLBCL cell lines**. In the figure, the left and the right sequence log representations of PBEMs are the predicted primary (inferred from type I SPIB binding sites) and the alternative (inferred from type II SPIB binding sites) SPIB binding motifs in ABC DLBCL cell lines, respectively, by using BayesPI2+.

From the above analysis, ChIP-seq peaks represent either type I or type II TF binding sites that can be distinguished via dbA and expected P-values. However, the question remains whether low affinity type II TF binding represents indirect binding or binding to alternative binding sites [10,21,22]. This question was investigated by searching for the putative TF binding motifs in the predicted type I and type II ESR1 and SPIB binding sites, respectively. A serial version of BayesPI2+ was first applied to find the best PBEM in 9657 type I and 7064 type II ESR1 binding sites, respectively. The results show that the known ESR1 binding motif is only enriched in ESR1 type I TF binding sites (Additional file 1: Figures S17 and S18). No ESR1 binding motifs were discovered in ESR1 type II TF binding sites. These results suggest that ESR1 type II binding represents indirect binding of ESR1. By contrast, the same analysis for SPIB shows that the SPIB core binding motif is similar for both type I and type II TF binding sites (Figure 9). However, there is a significant difference of the sequence at both 5' and 3' sides of the SPIB PBEMs. In particular, the core SPIB binding motif GGAA is followed by a G nucleotide in type I binding sites whereas it is followed by a C in type II SPIB binding sites. This single nucleotide replacement in the vicinity of the core SPIB binding motif may contribute to differential binding of SPIB in human genome. By applying the BayesPI2+ parallel ensemble approach on the same datasets for ESR1 and SPIB, after removal of low binding affinity sites, similar results were found (Additional file 1: Figures S18, S19, and S20).

### Verification of predicted type I and type II SPIB binding sites by *EMSA*

In order to verify SPIB binding sites, a two-step filtering procedure was applied to 6911 putative SPIB target genes ('A' genes) with SPIB binding motifs. For each putative SPIB target gene, a two-sample Kolmogorov-Smirnov goodness-of-fit hypothesis test was performed between time-series microarray gene expression profiles (lenalidomide treated Oci-ly10 or TMD8 cells) and the gene expression profile (HBL1 cells) after down-regulation of SPIB by shRNA [11,12]. The assumptions were that genes controlled by SPIB are differentially expressed (i.e. P-value of KSTest < = 0.05) in ABC DLBCL cells treated as the above-mentioned two conditions, and that SPIB regulated genes are those genes with either a type I or a type II SPIB binding sites located between 5 kb upstream and 1 kb downstream to the transcription start site. In total, 1687 genes fulfilled these criteria (Additional file 1: Table S3). To test whether those genes contained SPIB binding sites and whether type II binding sites represented alternative binding sites, as predicted by our analysis, EMSA was performed on 10 randomly selected type I SPIB binding sites and 10 type II SPIB binding sites (Additional file 1: Tables S4 and S5). For this analysis, 50bp DNA sequences were selected of which the center sequence corresponded with the identified peak in our analysis.

**Figure 10:**
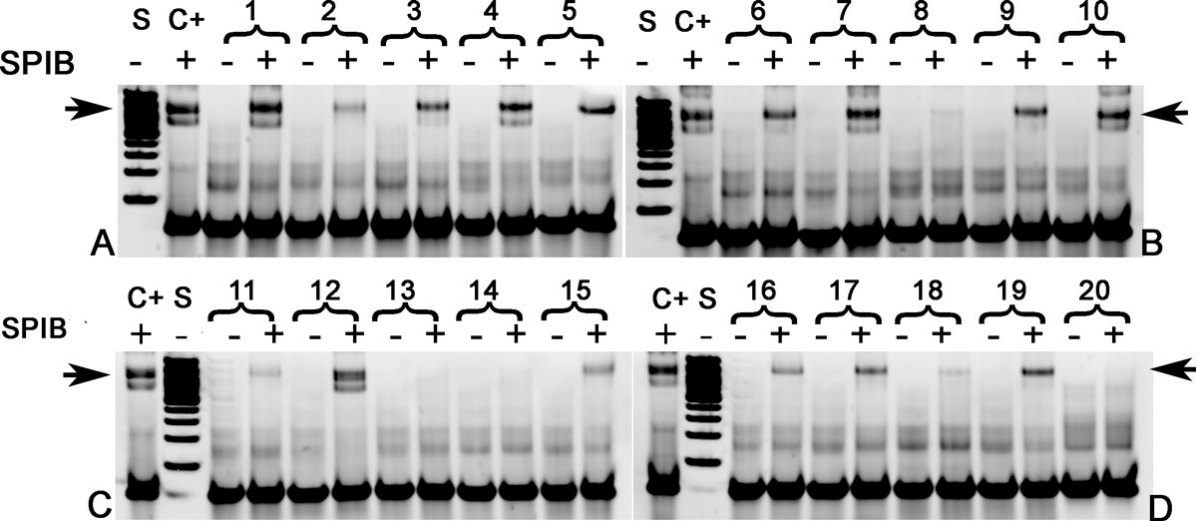
**Verification of type I and type II SPIB binding probes by EMSA with purified SPIB protein**. Gels A and B represent type I SPIB binding probes, gels C and D contain type II SPIB binding probes. SPIB means purified SPIB protein. The label of each column: C+ represents positive control of previously published SPIB binding probe [37], and - is the negative control, S is DNA size standard (lowest band 100bp, highest band 3000bp), and the number is the corresponding probe number in Additional file 1: Tables S4 and S5 for type I SPIB binding probes (i.e. 1 to 10) and type II SPIB binding probes (i.e. 11 to 20), respectively. The black arrow is pointing to the shifted band.

Of the 10 type I SPIB binding sequences, 8 showed very strong, one showed weak binding and one did not show binding. Of the 10 type II SPIB binding probes, 5 showed weak binding and 5 showed not binding (Figure 10). To check the binding to the SPIB core motif (GGAA), 2bp of the core SPIB motif were mutated (i.e. GGAA → TTAA) for two selected type I SPIB binding sites (Additional file 1: Table S6), then the same gel shift analysis were performed for both the original and the mutated type I SPIB binding sites. Additional file 1: Figure S21 showed that binding of SPIB is completely inhibited after mutating 2bp of the SPIB core motif. The results of these *in vitro *experiments support that type I and type II SPIB binding represents direct TF binding, with type II being, at least in part, alternative weak SPIB binding sites.

## Discussion

A new biophysical model, named BayesPI2+ was designed to analyze more extensively TF binding sites in *in vivo *protein-DNA interaction data than hitherto achieved with existing models. To achieve this BayesPI2+ was designed to analyze a large number of called ChIP-seq peaks (i.e. hundreds of thousands) simultaneously, including weak binding sites. Additionally, the differential binding affinity (dbA) for each called peak was computed to identify high affinity (type I) and low affinity (type II) TF binding sites. In this work, the strength of type I (direct) and type II (indirect/alternative) binding sites is not in proportion to the number of measured ChIP-seq tag counts. In other words, TF acts independently in type I (high affinity) binding but it requires a co-factor to stabilize the protein-DNA interaction in type II (low affinity) binding. An initial test was performed on synthetic ChIP-seq datasets with success, yielding a true negative rate of >90%. For the test, a serial version and a parallel ensemble learning of BayesPI2+ was used with similar prediction accuracies. However, in the test, the parallel version of BayesPI2+ is at least three times faster than the serial one in completing the prediction. Thus, for very large input data (i.e. more than ten thousands of called ChIP-seq peaks), a parallel ensemble learning of BayesPI2+ is recommended because it saves the overall waiting time considerably by splitting the job to multiple computer processors.

In a subsequent analysis of five human ChIP-seq datasets, the predicted PBEMs between a serial BayesPI2+ computation and a parallel ensemble learning of BayesPI2+ were compared. Though the best PBEM identified by serial computation and the meta-PBEM estimated by parallel ensemble learning were similar, a positional variation in information content of the predicted meta-PBEMs were observed across different sizes of input data (Figure 2 and Additional file 1: Figure S3). The positional variation in information content for PBEM reaches saturation point when the number of input peaks exceeds 25% of all called peaks. This indicates that various binding positions within a PBEM are important in the formation of the protein-DNA complex, during *in vivo *protein-DNA interactions [23,24]. By analyzing all possible binding sites with our method, two types could be discerned. Particularly, the dbA level of each TF, estimated by using the corresponding meta-PBEM, allowed to recognize low affinity TF binding, called type II TF binding sites (Figure 3 and Additional file 1: Figure S6). Additionally, the inferred meta-PBEMs from ChIP-chip data and the binding consensus sequence motifs from online databases [18] are significantly enriched in the type I TF binding sites when analyzing yeast ChIP-chip experiments for TFs ACE2, SWI4, and INO4. Of interest, the latter analyses were performed on ChIP-chip data, thus on genomic sequences of unequal length. Therefore, our proposed new method to distinguish two types of TF binding sites is applicable to both equal-length and unequal-length genomic sequences. Of note, BayesPI2 has a very good agreement (> 80% overlap) with CentriMo concerning the analysis of type I (direct) TF binding.

To test the function of predicted type I and type II TF binding sites, we analyzed their effect on gene regulation. This was done for two human TFs, ESR1 and SPIB in breast cancer and diffuse large B cell lymphoma, respectively. ESR1 encodes the estrogen receptor alpha and contributes to cell growth in breast carcinoma [11]. SPIB is a B cell transcription factor that is highly expressed in a clinically aggressive subtype of DLBCL, with activated B cell immunophenotype [12]. Genes with the putative TF binding sites were classified into three groups: 'A' genes, containing both type I and type II TF binding sites; 'B' genes containing only type I TF binding sites; and 'C' genes, containing only type II TF binding sites. Of interest, 'A' genes show the strongest expression in the breast cancer cell line MCF7 and the highest ESR1 ChIP-seq tag density. In addition, we found lower nucleosome density, higher RNA polymerase II expression and histone acetylation for 'A' genes than for 'B' or 'C' genes. It is known that decreased nucleosome density and increased RNA Pol-II binding indicate gene transcription [25,26] whereas acetylation of histones (i.e. H3K14ac, and H3K9ac) indicate functional TF binding [25] and are important for activation of promoters and enhancers [27]. Functional annotation of the three types of ESR1 target genes also suggests that, only 'A' genes are highly enriched in pathways of importance to breast cancer such as the MAPK signaling pathway [28]. Analogous to what is observed for ESR1 in breast carcinoma, SPIB 'A' genes show the highest gene expression in diffuse large B cell lymphoma, and the strongest response after silencing of SPIB by RNA interference in ABC DLBCL cell lines. Functional annotation of SPIB 'A' genes suggests that they are significantly involved in pathways of importance for DLBCL lymphoma biology, such as the B cell receptor signaling pathway. The B cell receptor signaling pathway is activated in diffuse large B-cell lymphoma and contributes to tumor cell survival and proliferation [29]. Thus, our results indicate that ESR1 and SPIB regulation of gene expression requires both type I and type II binding. A repeat analysis of genes controlled by either ESR1 or SPIB binding sites, after filtering out the lowest binding affinity sites, yielded similar results: 'A' genes are the likely functional targets of these TFs.

Though the effect of TF on genes requires direct protein-DNA interaction, it has been well-known that additional and different interactions of the TF with the gene are needed for regulation [30]. We show for the first time the scale of this effect by our genome-wide analysis [1,31]. Type II TF binding sites for ESR1 and SPIB were further investigated to find out whether these constituted indirect binding sites or alternative binding sites with different affinity. No ESR1 similar motif was found for type II ESR1 binding sites. This indicates that type II ESR1 likely represents indirect binding to DNA. By contrast, a similar consensus motif was found for both type I and type II SPIB binding sites, except for one nucleotide difference in the area adjacent to core SPIB motif GGAA (Figure 9). These results indicate that the type II SPIB binding site is an alternative low affinity SPIB binding site. The findings were also confirmed by EMSA: about 90% and 50% of tested sequences with putative type I and type II SPIB binding sites, respectively, resulted in band shift in EMSA (Figure 10). These results also confirm that type II TF binding is rather weak, which requires co-factors to enhance the binding to DNA sequence. Notwithstanding, our analysis shows that type I and type II binding sites act concertedly in gene regulation.

## Conclusions

In summary, we developed a new program BayesPI2+ to analyze all potential TF binding sites in the genome, without filtering. By doing so, we found two types of functional TF binding sites, both indicate to be important for gene regulation. BayesPI2+ can be used in a serial computation or a parallel ensemble approach. Hitherto, other methods only used the top few hundred or thousand high affinity binding sites which we showed results in loss of valuable information.

## Methods

### Synthetic ChIP-seq datasets

Four synthetic ChIP-seq datasets were generated by the Monte Carlo sampling method [8]. Each dataset has 500 putative target sites with DNA sequence length 500 bp. One of four yeast TFs (i.e. ACE2, SWI4, INO4 and XBP1 with binding motif length of 6 bp, 8 bp, 10 bp and 12 bp, respectively) was randomly positioned in a DNA sequence with a synthetic Z-score greater than 0.25. The synthetic Z-scores were produced by the MATLAB build-in random number generator. The synthetic ChIP-seq datasets were used to evaluate prediction accuracy of BayesPI2+ because type I (direct) binding targets can be easily recovered later. The final prediction accuracy of each synthetic ChIP-seq data is shown in box plots, where the error rates are estimated by randomly splitting the data to training (400 target sites) and test sets (100 target sites) five times.

### Human ChIP-seq datasets

Called peaks of ChIP-seq experiments for human CTCF in CD4+ T cell, NRSF in Jurkat T lymphoblast cell, and STAT1 in interferon y-stimulated Hela S3 cell were obtained from previous publications [13], where raw reads were mapped to hg18 reference genome. There are around 5814, 26815 and 73957 called peaks by SISSRS [13] for NRSF, CTCF, and STAT1, respectively. Raw ChIP-seq reads of human ERα in breast cancer cells (MCF-7) under both E2 treated and control conditions, and human SPIB in ABC DLBCL cell line HBL1 and control condition were downloaded from earlier publications [12,14]. These raw reads were aligned to hg19 reference genome by BWA program [32], and the peaks were called by SISSRS (i.e. 16720 and 43135 peaks for ERα and SPIB, respectively). For a particular TF ChIP-seq experiment under a specific condition or cell lines, both DNA sequences of putative protein binding sites and the corresponding tag densities were used by BayesPI2+ to infer protein binding energy matrix (PBEM) and the chemical potential. For each binding site, a 200 bp DNA sequence centered on the genomic coordinate of called peak was extracted from reference genome. GREAT [33] tool was used to assign protein binding sites to the putative target genes. The assignment of TF binding sites with nearby genes is a two step process: first, every gene is assigned a basal regulatory domain (i.e. 5kb upstream and 1kb downstream of the transcription start site); then, the gene regulatory domain is extended in both directions to the nearest gene's basal domain but no more than the maximum extension (i.e. 1000 kb) in one direction.

### Mouse ChIP-seq data sets

Three mouse embryonic stem cell (ES) TF ChIP-seq data sets (STAT3, MYC, and OCT4/POU5F1) were downloaded from a previous publication [34]. DNA sequence centered on each called peak (500 bp) and the normalized tag counts are obtained from papers [7] and [34], respectively.

### Human histone modification and microarray gene expression datasets

Histone modification datasets (e.g., H3K14ac, H3K27me3, H3K4me1, H3K4me2, H3K4me3, H3K9ac, H3K9me2 and H3K9me3), DNA accessible regions (FAIRE), and Pol-II data under both E2 treated and control conditions in MCF-7 cells were obtained from previous publications [14,19,20]. Pre-processing of the datasets and enrichment tests (*t*-test and Mann-Whitney *U *test) between E2 treated and control conditions were described in detail in previous studies [25,26]. Microarray gene expression profiles for E2 treated MCF-7 cells, lenalidomide induced ABC DLBCL cells and shRNA transduction treated SPIB in HBL1 cells are taken from published works [11,12]. The DAVID tool [35] was used to perform functional analysis of identified protein target genes.

### Yeast ChIP-chip datasets

Genome-wide *in vivo *protein-DNA interaction datasets of 4 yeast *S. cerevisiae *TFs (ACE2, SWI4, INO4 and XBP1) in rich medium conditions, the corresponding intergenic DNA sequences, and the P-values of significance of intergenic DNA sequences bound by each given TF were obtained from the work of Harbison et al. [16]. In the original paper, p < 0.001 was used to determine DNA regions were bound by a TF. Here, a confidence level p < 0.1 is used to select putative TF binding targets (i.e. intergenic DNA sequences with either low or high affinity TF binding sites) that need to be classified into type I and type II binding targets for each given TF, respectively.

### Biophysical model to distinguish type I versus type II TF-DNA interaction

#### Protein-DNA binding probability

Following previous descriptions of biophysical modeling of protein-DNA interactions [8,36], the probability of a DNA sequence *S *to be bound by a protein is $P\left(S\right)=\frac{1}{1+\text{exp}\left(E∙S-\mu \right)}$, where *E *represents PBEM (i.e. the estimated binding energy at each nucleotide; protein binding energy matrix) and *μ *is the concentration of proteins (or chemical potential) in a solution. This is a Femi-Dirac form of protein binding probability. If the concentration of protein is very low then the Fermi-Dirac function can be approximated by a Maxwell-Boltzmann protein binding function [37]$P\left(S\right)\approx \text{exp}\left(-E∙S\right)$. In earlier works, BayesPI [8] and MatrixREDUCE [38] had used Femi-Dirac form and Maxwell-Boltzmann function to model genome-wide *in vivo *protein-DNA interaction experiments, respectively, by combining measured TF occupancy data and PBEM prediction for each TF.

#### Computational implementation of BayesPI2+

BayesPI2+ is a pure C program with several build in functions: for example, various normalization options for the input data, DNA strand specific calculation, DNA binding affinity calculation based on PBEM, chemical potential estimation by using known PBEM, and prediction of PBEM with multiple motif length in parallel etc. In this work, both a serial computation (similar to original BayesPI MATLAB program[8]) and a parallel ensemble learning approach (estimating meta-PBEM; mean of multiple predictions) are developed. Prediction of PBEM from measured high-throughput sequencing data is achieved by a Bayesian Hierarchical nonlinear regression model [8], where a Fermi-Dirac form of protein binding probability was adopted. Detailed description of the algorithm is available in the earlier works [8,39], which is further improved in the serial version of BayesPI2+. The new parallel ensemble learning of BayesPI2+ is accomplished by a combination of C and Perl programs, where the C program is used to perform nonlinear parameter fitting and Perl scrip files are utilized to perform random selection of input data, split data to multiple computer processes, calculate motif similarity scores [17], and obtain a meta-PBEM by aligning multiple predicted PBEMs.

#### Estimation of meta-chemical potential

In BayesPI2+, a new function is added to estimate TF chemical potential (TF concentration *μ*) for known PBEM, where the PBEM of protein binding probability *P(S) *is fixed but the TF chemical potential *μ *is estimated from *in vivo *protein-DNA binding data by Bayesian nonlinear parameter fitting. In this way, TF meta-chemical potential is calculated based on a given meta-PBEM. Generally, an ensemble learning of meta-PBEM, by randomly drawing a portion of input data multiple times in parallel, is more robust than a serial prediction of PBEM from one data set. That is because the input data quality (outliers) may reduce the robustness of nonlinear parameter fitting for both PBEM and chemical-potential [9]. In addition, if the input data size is large then the nonlinear parameter fitting will suffer significantly from a long serial computation. Thus, the parallel ensemble learning will not only speed up the calculation, but also improve the prediction accuracy.

#### Calculation of TF binding affinity with either Femi-Dirac or Maxwell-Boltzmann function

The second new function in BayesPI2+ is TF binding affinity estimation, which is given by $Y_{i}=w\overset{N-m+1}{\underset{l=1}{\sum }}P_{i,l}\left(S_{i}\right)$, $S_{i}$represents a DNA sequence, *N *is the length of the sequence, *m *is the length of TF binding site, *w *is a weight coefficient and equals one, and $P_{i,l}\left(S_{i}\right)$ is the protein-DNA binding probability. If TF chemical potential is included in the calculation then Femi-Dirac form of protein binding probability will be used by $P_{i,l}\left(S_{i}\right)$. Otherwise, Maxwell-Boltzmann function will be chosen in $P_{i,l}\left(S_{i}\right)$ if information of TF chemical potential *μ *is not available. The latter implementation is equivalent to the previous work of matrixREDUCE [40]. In this work, if both PBEM and chemical potential are inferred from high-throughput protein-DNA interaction data, then the Femi-Dirac function is used to compute TF binding affinity. For consensus sequence motifs that collected from on-line databases, Maxwell-Boltzmann function will be used to estimate the TF binding affinity.

#### Computation of differential binding affinity - dbA

For each TF, its protein binding affinity $Y_{i}$ to a DNA sequence *S_i _*(i.e. 200bp DNA sequence centered on the called peak) is computed based on the above-mentioned description. Differential binding affinity (dbA) to the DNA sequence *S_i _*is defined as $dbA_{i}=Y_{i}-\frac{\overset{R}{\underset{r=1}{\sum }}Y_{i,r}}{R}$, where $Y_{i,r}$ represents estimated protein binding affinity at r^th ^randomly mutated DNA sequence *S_i_*, and *R *is the total number of random shuffling of DNA sequence *S_i_*,. Expected P-value of *dbA_i _*to a sequence *S_i _*is the ratio between the number of $Y_{i,r}\geq Y_{i}$ and the total number of random shuffling. Usually, if a DNA sequence *S_i _*contains a protein binding motif (type I TF binding) then $Y_{i,r}<Y_{i}$. On the contrary, if there is not a direct protein binding motif (type II TF binding) in the DNA sequence *S_i _*then $Y_{i,r}\geq Y_{i}$. In the latter case, the type II protein-DNA interaction is treated as a protein interacts to randomly mutated DNA sequences (i.e. DNA sequence of each protein binding site is randomly shuffled *R *times). Generally, the smaller the P-value the better the type I TF-DNA binding, the larger the P-value the better the type II TF-DNA binding. In this work, these calculations are split to multiple computer processes and run in parallel, which significantly reduces the overall waiting time.

*A serial computation*. Using a serial version of BayesPI2+ to distinguish type I versus type II protein-DNA interactions: 1) to predict the best representative PBEMs with various lengths for all called peaks; 2) to calculate motif similarity scores [8] between the predicted PBEMs and a golden standard one (i.e. a position specific weight matrix (PSWM) from either JASPAR [41] or TRANSFAC [42]), and a PBEM with the highest motif similarity score is selected; 3) to compute protein binding affinities for all called peaks by using the above-chosen PBEM and its chemical potential; 4) to calculate *dbA *for all called peaks based on the same PBEM, where 200bp DNA sequences that centered on each peak are randomly shuffled 2000 times; 5) to compute expected P-value of *dbA *for all called peaks (the expected chance of type I binding at a target site); 6) to classify all called peaks to two groups (i.e. type I and type II protein-DNA interactions) by applying fuzzy neural gas algorithm [31,43] (Additional file 1: supplementary methods) on the *dbA*, where the classification between type I and II TF binding can be further improved by adding expected P-values (i.e. for human TFs, type I TF bindings with expected p < 0.09). It is worth noting that *dbA *may reveal the true protein binding pattern in different genomic regions because the effect of variation of background binding is removed.

#### A parallel ensemble learning framework

Though BayesPI2+ can handle a large number of called peaks in one run, the computational cost is increased significantly when the number of input peaks reaches hundreds or thousands. In order to avoid such hindrance by the big data, a parallel ensemble learning version of BayesPI2+ is built: 1) to randomly select a subset of all called peaks (i.e. 25%), the random selection is repeated multiple times (i.e. 10 times); 2) to estimate PBEM and the corresponding parameters based on each randomly selected subset, for example, ten computer processes control ten randomly selected subsets and run step 1 of the serial BayesPI2+ computation in parallel; 3) to compute motif similarity scores between all predicted PBEMs and a golden standard one (i.e. a PSWM from JASPAR), and to obtain a meta-PBEM by aligning good PBEMs (i.e. motif similarity scores >0.7) against the golden standard one; 4) to infer meta-chemical-potential for the new meta-PBEM based on all called peaks; 5) to calculate expected P-value and *dbA *for all called peaks based on inferred meta-PBEM and meta-chemical potential (i.e. steps 3, 4, and 5 of the serial BayesPI2+ computation); 6) to classify all called peaks to two groups (i.e. type I and type II protein-DNA interactions) by applying fuzzy neural gas algorithm on *dbA*, and the classification between type I and II TF binding can be further improved by using expected P-values (i.e. for human TFs, type I TF bindings with expected p < 0.09). All calculations were done on the Linux cluster, where computer nodes have a minimum 64 GB RAM, and 16 physical CPU cores and are connected by FDR (56Gps) InfiniBand.

### Electrophoretic mobility shift assay (EMSA) for detecting protein-DNA interactions

EMSA [37] was performed with the BioRad Mini Protean gel system (BioRad, USA) at 90V for 1 hour. The binding reactions were performed for 30 minutes with the Odyssey™ EMSA Buffer Kit (LI-COR, USA) according to the manufacturer recommendations with some modifications. Binding reaction: 1x binding buffer, 2.5mM DTT/0.25% Tween20, 2.5% glycerol, 8ng/μl of SPIB protein, 250nM of probe and a total incubation volume of 20 μl. Products were resolved by polyacrylamide gel electrophoresis using a 10% Mini-PROTEAN^® ^TBE Precast Gel (BioRad), and 0.5 × TBE buffer, then analyzed by staining with GelRed Nucleic Acid Gel Stain, (Biotium, USA) and visualized by UV lamp. Purified SPIB (Human) Recombinant Protein (P01) was purchased from Abnova (Taiwan). Double stranded (ds) 50bp DNA probes were annealed from ssDNA oligonucleotides in 1xTE buffer pH 8 with addition of 50mM NaCl, final probe concentration was 1.5 μM. Probes were heated to 90^o^C for 5 min, then slowly cooled down to RT in 2h. The 10% TBE gels were prerun in 0.5x TBE buffer for 1h at 90V. Specificity of binding was demonstrated by mutation of the putative SPIB core binding motif in two probes, GG was changed to TT (GGAA→TTAA).

## Competing interests

The authors declare that they have no competing interests.

## Authors' contributions

JBW and JD conceived study. JBW designed the study, implemented program in BayesPI2+, performed data analysis and results interpretation, and drafted manuscript. AM and GT designed and performed EMSA experiment. JD participated in writing part of manuscript. All authors participated in results discussion, read and approved the final manuscript

## Supplementary Material

Additional file 1 **Additional Figures and Tables** Contains all additional tables and figures. Click here for file

## Declarations

The publication costs for this article were funded by Norwegian Cancer Society (DNK 2192630).

This article has been published as part of *BMC Genomics *Volume 16 Supplement 7, 2015: Selected articles from The International Conference on Intelligent Biology and Medicine (ICIBM) 2014: Genomics. The full contents of the supplement are available online at http://www.biomedcentral.com/bmcgenomics/supplements/16/S7.
