# Supplementary material for: Comprehensive genome-wide transcription factor analysis reveals that a combination of high affinity and low affinity DNA binding is needed for human gene regulation
Source: BMC Genomics. 2015 Jun 11;16(Suppl 7):S12. doi: 10.1186/1471-2164-16-S7-S12 (PMC4474539; doi:10.1186/1471-2164-16-S7-S12)
Supplement: Additional file 1 — Additional Figures and Tables Contains all additional tables and figures. [file 1471-2164-16-S7-S12-S1.doc]

**Supplementary to ‘Comprehensive genome-wide transcription factor analysis reveals that a combination of high affinity and low affinity DNA binding is needed for human gene regulation’**

**Junbai Wang, Agnieszka Malecka, Gunhild Trøen and Jan Delabie**

**Supplementary Methods**

***Fuzzy neural gas algorithm*** *(FNG).* FNG is a combination of fuzzy logic and neural gas algorithm [1], which uses a similar “soft-max” adaptation rule as maximum-entropy clustering and self-organizing maps (SOMs) to summarize high-dimensional input space (i.e. ChIP-seq called peaks) to low-dimensional reference vector space (i.e. two types of TF-DNA interactions). Then, the input data is assigned to the nearest class prototype and the fuzzy membership estimates the confidence level of the classification. Though many other machine-learning methods can be utilized to perform the same task, the Fuzzy neural gas algorithm is capable of performing unsupervised learning and capturing nonlinear relationships between the features and sample classes.

**Supplementary Results**

**Enrichment test of collected yeast TF consensus binding motifs in predicted type I and type II TF binding sites**

To further verify predictions from three yeast ChIP-chip experiments, a set of yeast transcription factors sequence motifs (i.e. ~136 yeast TFs with 234 TF consensus sequence motifs) were collected from both SGD and YEASTRACT databases [2]. BayesPI2+ was used to compute TF binding affinity of each consensus motif on type I TF binding sites, type II TF binding sites, and the rest of intergenic regions for ACE2, SWI4 and INO4, respectively. Subsequently, a t-test was used to evaluate enrichment of each consensus motif on either type I TF binding targets or type II TF binding ones. T-values of all tested consensus sequence motifs are shown in SFigure 9, the top 50 enriched yeast consensus sequence motifs on type I and type II TF targets are displayed in SFigure 10. The results are consistent with the previous finding: for example, in SFigure 10, consensus sequence motifs of the three selected yeast TFs are not enriched in the type II TF binding targets; however, the consensus sequence motifs of ACE2, SWI4 and INO4 are among the top 3 most enriched protein binding sites at their type I TF binding targets, respectively; additionally, protein binding complex or cofactors of the yeast TFs are appeared in the top 3 enrichments (i.e. INO2-INO4 complex). Taken together, the classification of type I and type II TF binding targets in three yeast ChIP-chip experiments is good, and the proposed new biophysical model (BayesPI2+) is suited for distinguishing type I verse type II protein binding targets in unequal-length genomic sequences.

**Functional annotation of three types of putative ESR1 target genes by using DAVID**

Furthermore, a functional annotation of three classes of ESR1 targets was performed by using DAVID tool. Table 1 shows that ESR1 targeted ‘A’ genes are highly enriched in many pathways in cancer (98 genes) such as MAPK signaling pathway and Insulin signaling pathway; ESR1 regulated ‘B’ genes show only marginal in Calcium signaling pathway (50 genes); however, ESR1 targeted ‘C’ genes are not enriched in any pathways. For tissue expression analysis, only ESR1 ‘A’ genes are frequently linked to mammary gland grade I ER+, PR+, Her2- invasive ductal carcinoma 3rd (180 genes); mammary gland invasive breast cancer ER+, PR+, Her2-, grade II 3rd (270 genes); and mammary gland ductal carcinoma in situ, extensive, grade III, Her2+_3rd (180 genes) etc.,. ESR1 ‘B’ genes show enrichment in diverse cancer tissues such as prostate adenocarcinoma 3rd (113 genes), prostate_carcinoma_3rd (92 genes) and white blood cells invasive breast cancer, ER+, PR+, Her2- 3rd (70 genes). However, ESR1 ‘C’ genes are mostly associated to brain and stem cell (i.e. brain anaplastic astrocytoma grade III, 274 genes; spinal cord normal spinal cord 3rd, 391 genes; and stem cell null 3rd, 261 genes). In summary, functional ESR1-DNA interactions may be occurred at ESR1 ‘A’ genes because of the supporting evidences from diverse information (i.e. SFigure 11, Figures 5, 6 and Table 1).)

**Supplementary Figures**

**SFigure 1 Error rates of distinguishing type I versus type II TF binding sites in synthetic ChIP-seq data sets by using a parallel ensemble version of BayesPI2+.**

**
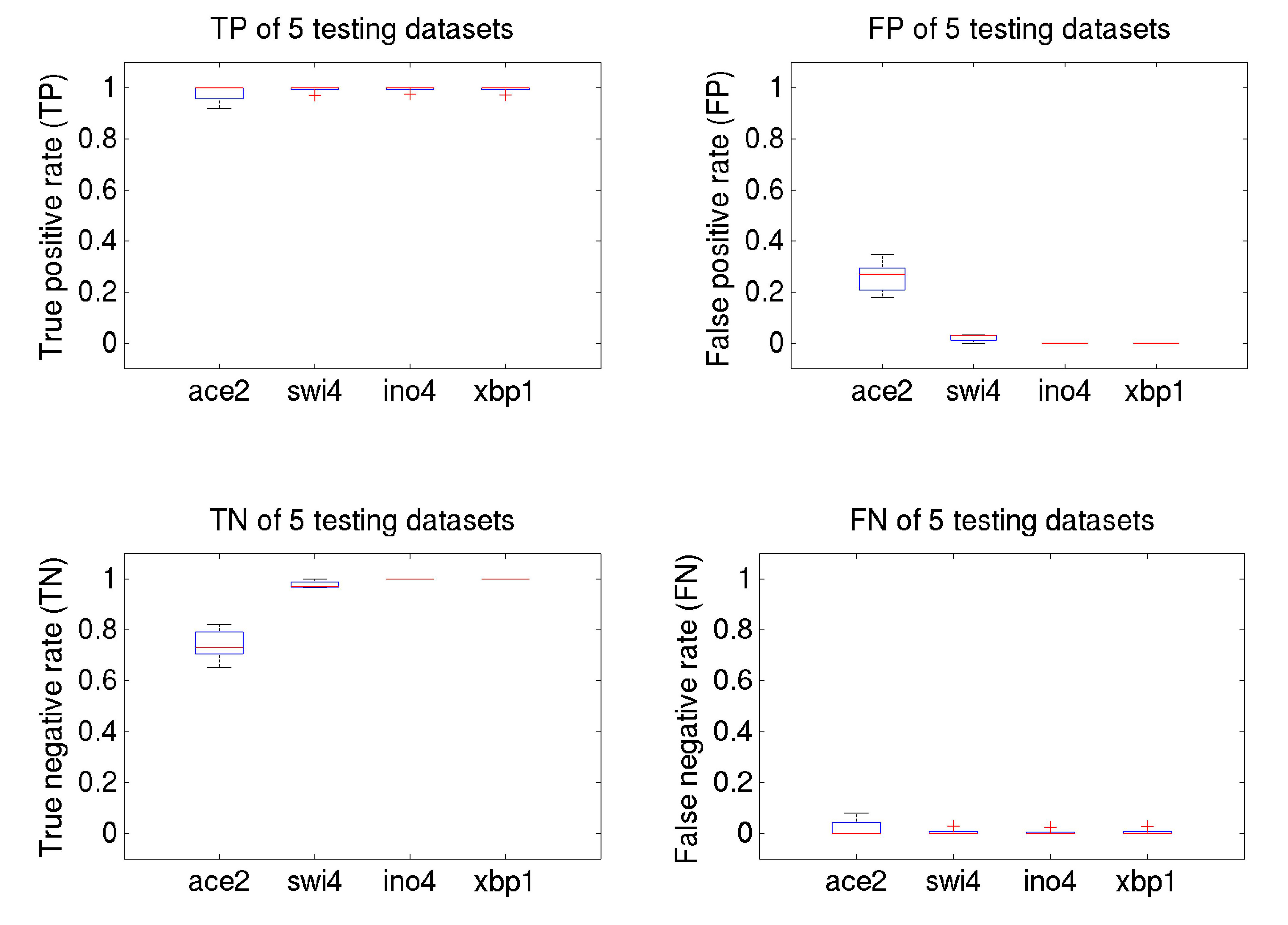
**

Error rates (i.e. TP – true positive rate, FP – false positive rate, TN – true negative rate, and FN – false negative rate) are displayed in box plot, which were estimated by randomly splitting training and testing data five times, and the inferred meta-PBEM from the training data was used to compute dbA and to distinguish type I versus type II TF binding sites in the testing data.

**SFigure 2. CPU time used by BayesPI2+ to learn PBEMs based on various input sizes of called peaks.**

**
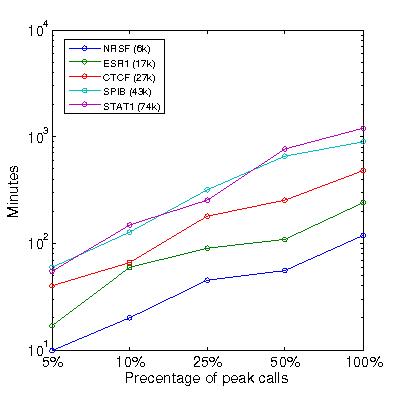
**

Here, a parallel ensemble learning of BayesPI2+ was used to infer meta-PBEMs for each human TF, by randomly selecting (10 times) a portion (i.e. 5%, 10% 25%, and 50%) of all called peaks; and a serial version of BayesPI2 was applied on 100% of all called peaks to infer the best PBEM for each TF. The computational cost (CPU hours) of the above-mentioned calculations are recorded in the figure.

**Sfigure 3. Comparisons of predicted PBEMs based on various input sizes
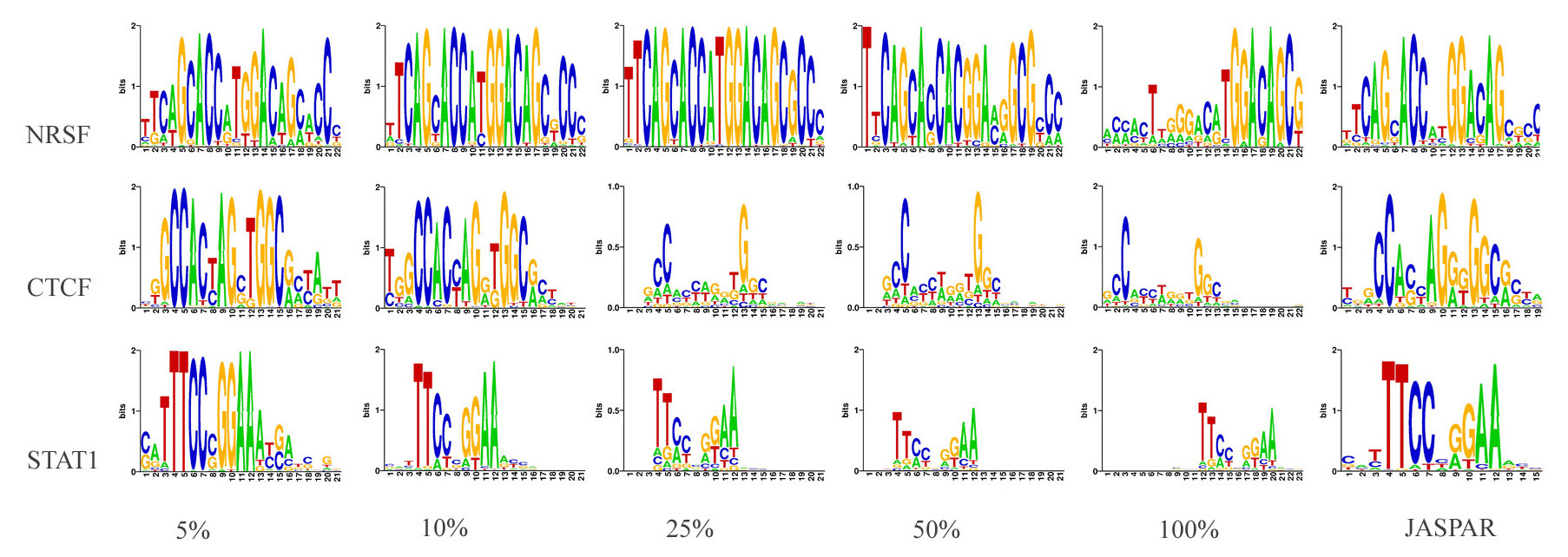
**

A parallel ensemble learning and a serial computation of BayesPI2+ was applied on a portion of randomly selected (i.e. 10 times random selections of 5%, 10%, 25%, 50% of the peaks) and all (i.e. 100%) called peaks, respectively. Sequence log representations of the best representative PBEMs (100% called peaks) and the meta-PBEMs of various sizes of input data are shown in the figure. The last column is the known NRSF, CTCF and STAT1 binding motifs from JASPAR database.

**Sfigure 4 Predicted meta-PBEMs of five human TFs by applying a parallel ensemble version of BayesPI2+ on 25% of all called peaks.**

**
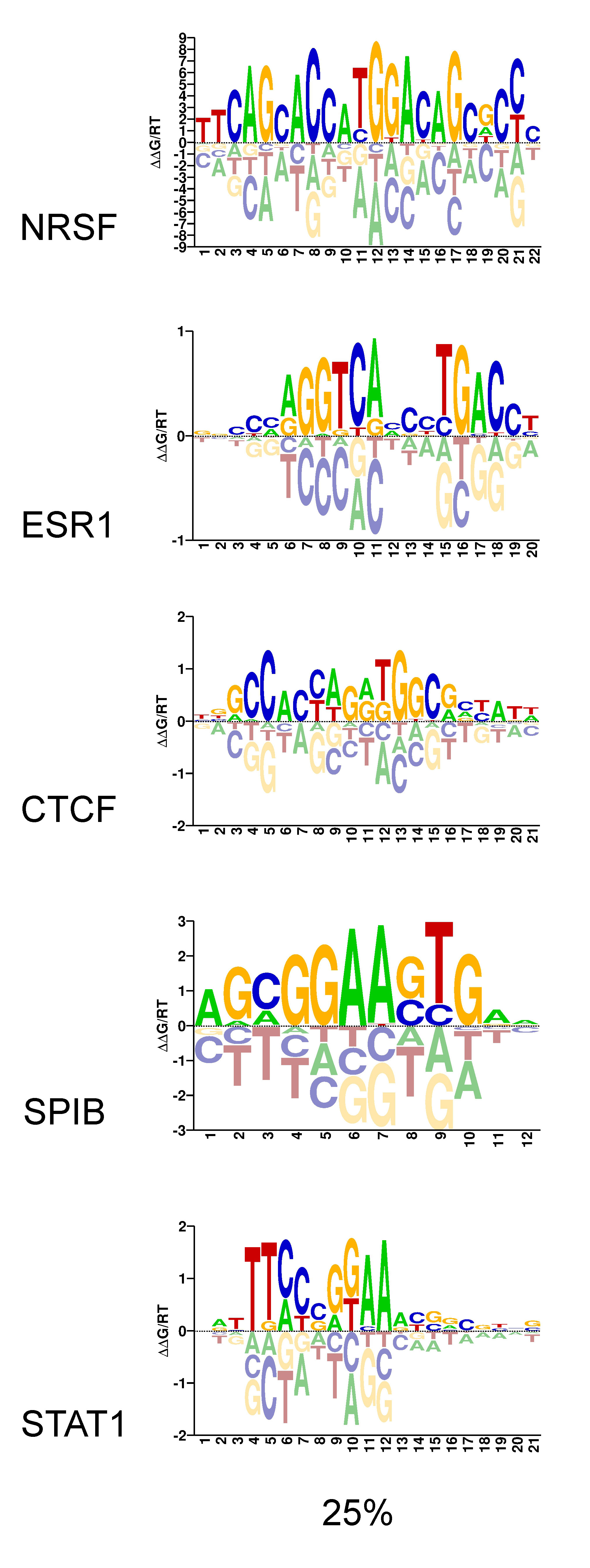
**

Here, meta-PBEMs (positive, negative binding energy) for five human TFs are illustrated. The meta-PBEMs were inferred by applying a parallel ensemble learning of BayesPI2+ on randomly selected 25% of called peaks 10 times. For PBEMs with low information content (i.e. ESR1, CTCF, and STAT1), PBEMs seem to have better illustration of position specific binding energy than those sequence log representations in Figure 2 and SFigure 3.

**SFigure 5 Motif enrichment test at predicted type I and type II TF binding sites for five human TFs.**


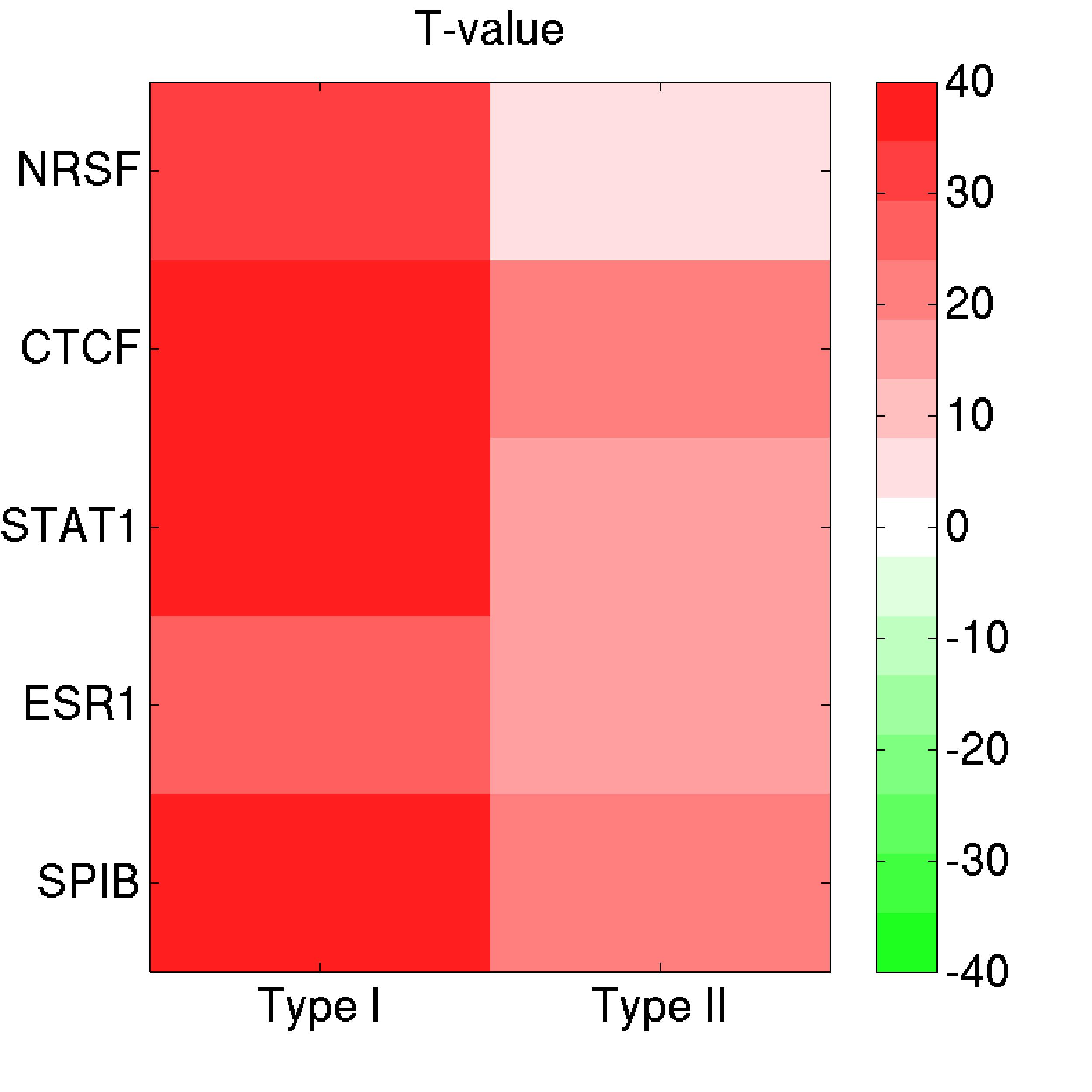


In the figure, “type I” and “type II” means predicted two types of protein binding sites, respectively. Here meta-PBEMs were estimated by applying a parallel ensemble learning of BayesPI2+ on randomly selected 25% of called peaks 10 times. These meta-PBEMs were used to compute dbA and to classify type I versus type II protein binding sites. Enrichment of meta-PBEMs in type I and type II TF binding sites is evaluated by a linear regression model (i.e. fitting the predicted TF binding affinities to the measured ChIP-seq tag density at all protein binding sites). Then the regression coefficient was transformed to T-value for accessing the significance of the predicted meta-PBEM in either type I or type II TF binding sites.

**Sfigure 6** **Distribution of ChIP-seq tag density and differential binding affinity (dbA) at predicted type I and type II TF binding sites.**

**
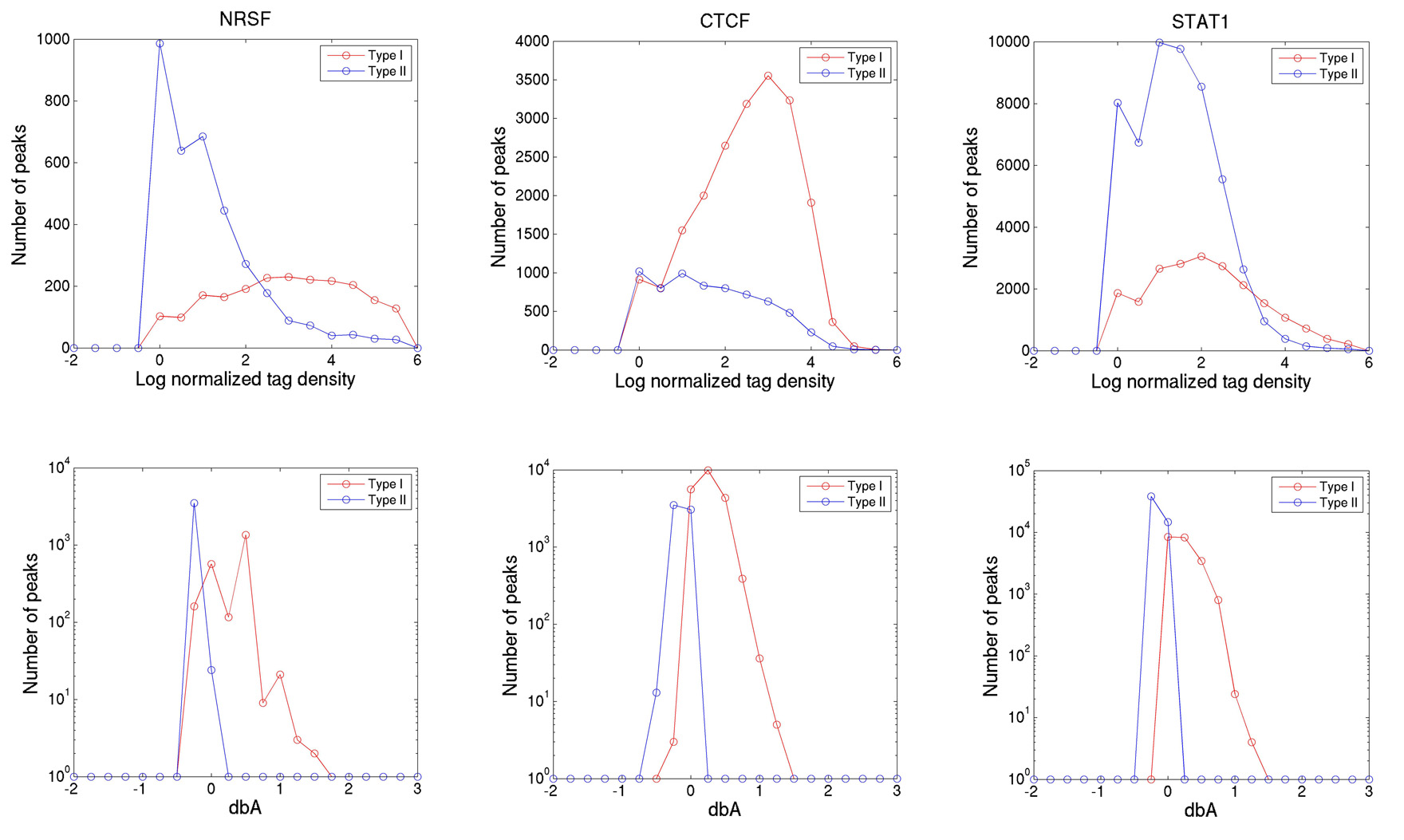
**

By random drawing 25% of called ChIP-seq peaks ten times, a parallel ensemble version of BayesPI2+ was used to obtain meta-PBEMs of human NRSF, CTCF and STAT1, respectively. Then, the meta-PBEMs were used to compute expected P-values and dbA, and to classify type I and type II TF binding sites. In the figure, left, middle and right panels illustrate distributions of log normalized ChIP-seq tag density, dbA level of type I (red lines) and type II binding sites (blue lines) for NRSF, CTCF, and STAT1, respectively.

**SFigure 7 Sequence log representation of the predicted best representative PBEMs from three yeast ChIP-chip experiments.**

**
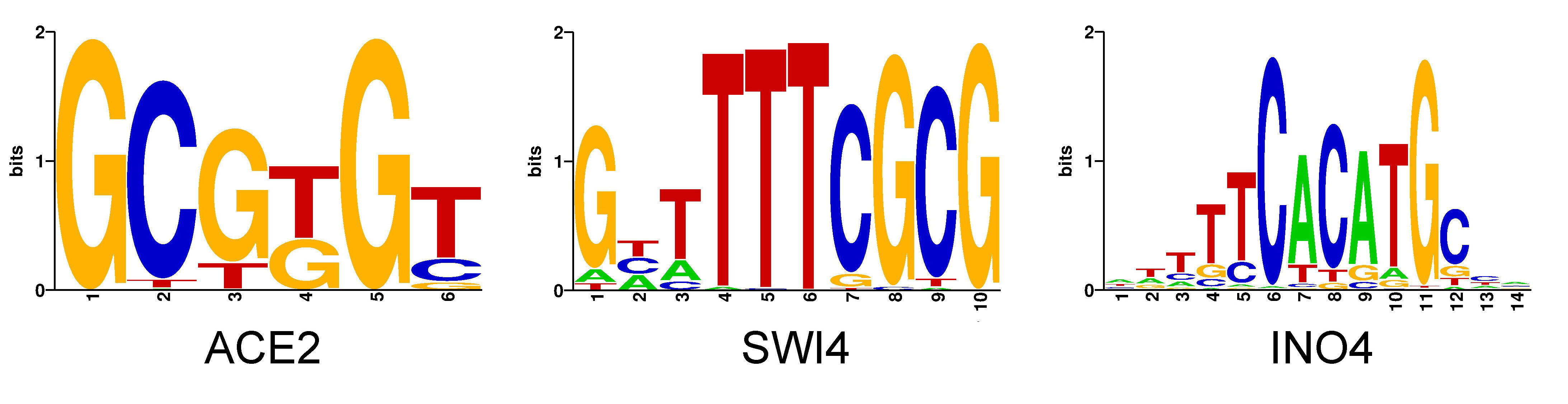
**

A serial version of BayesPI2 was applied on three yeast ChIP-chip (ACE2, SWI4, and INO4) experiments in rich medium condition. The best representative PBEM to each prediction is illustrated in the figure, respectively. Here, the protein binding sites are located on ~6725 unequal-length yeast intergenic regions.

**SFigure 8 A plot of protein binding confidence level versus the number of protein binding sites above that confidence level.**

**
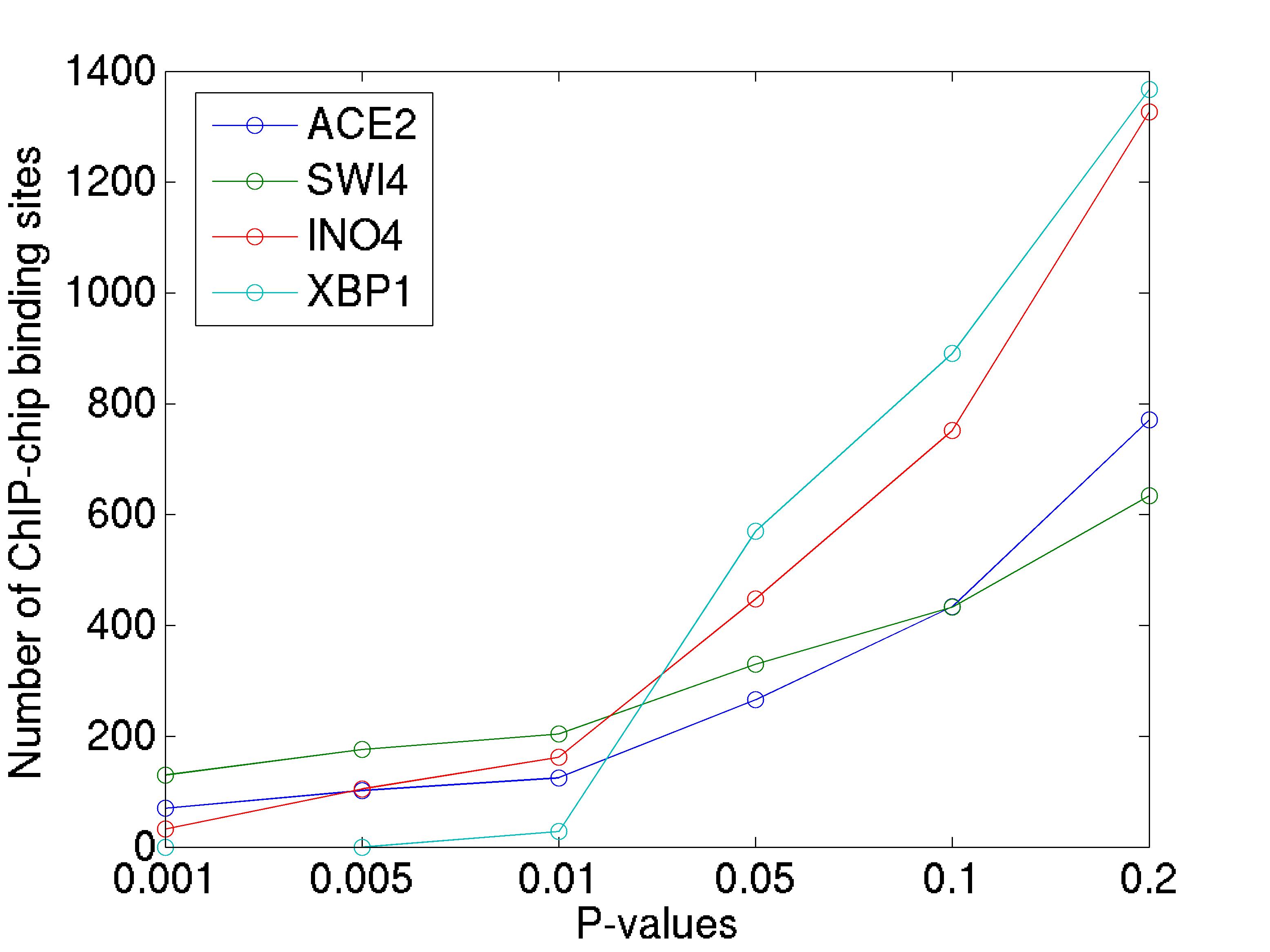
**

In the figure, X-axes is a P-value to the confidence level of protein binding sites from ChIP-chip experiments [3], and Y-axes is the number of protein binding sites with a confidence level above that P-value in the X-axes.

**Sfigure 9. Enrichment test of yeast protein binding motifs at predicted type I and type II protein binding sites.**

**
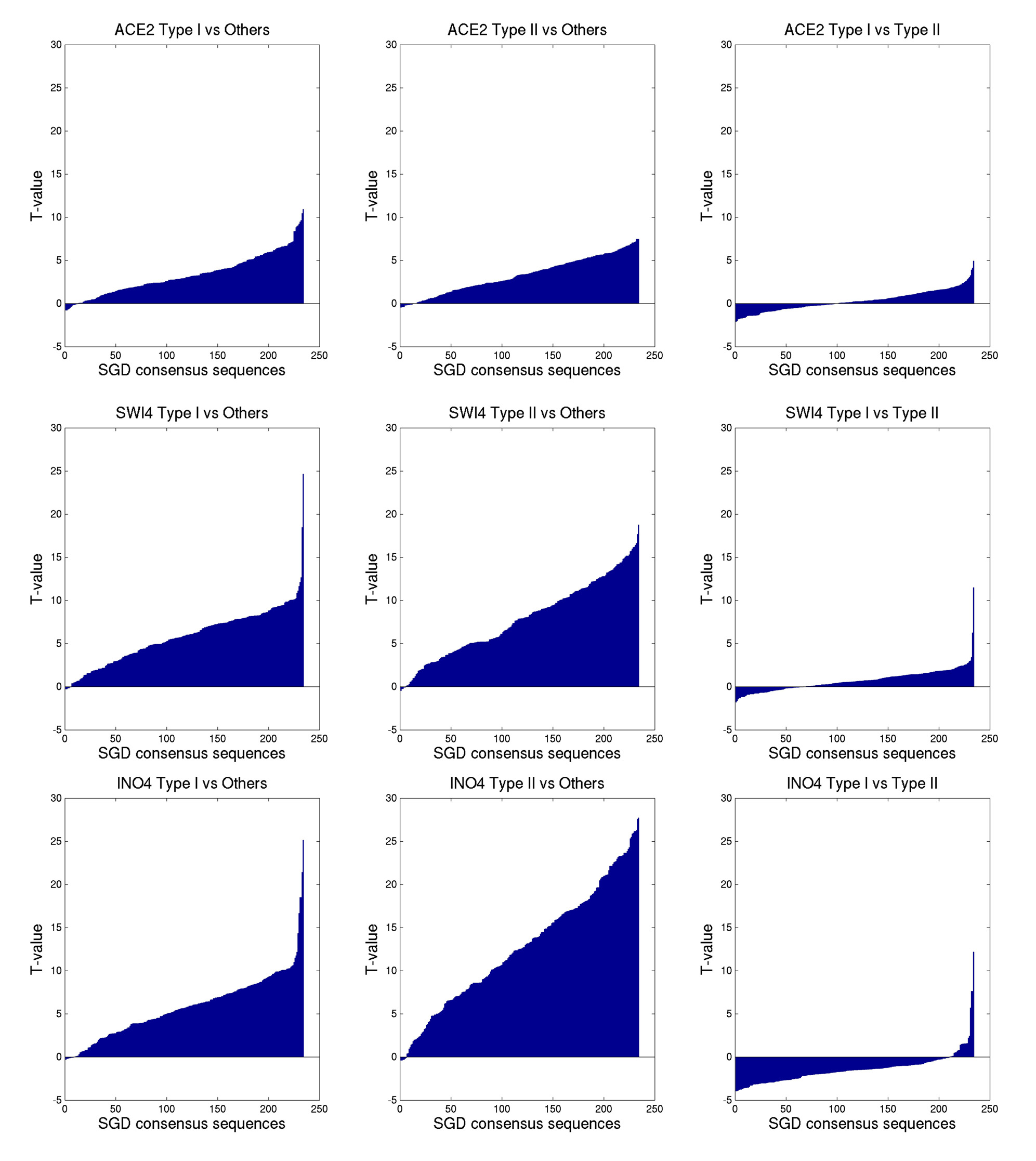
**

In the figure, “type I” represents predicted type I (direct) protein binding targets, “type II” means predicted type II (indirect/alternative) protein binding targets, and “Others” represents the rest of yeast intergenic regions in ChIP-chip experiments. Here, 234 yeast TF consensus sequence motifs were collected from SGD and YEASTRACT database, and BayesPI2+ was used to compute TF binding affinity of each consensus motif on type I, type II, and the rest of integenetic regions for ACE2, SWI4 and INO4, respectively. Enrichment of the consensus motifs in the above-mentioned three types of TF binding sites is evaluated by a two-tailed t-test (i.e. type I vs. Others, type II vs. Others, type I vs. type II), and the T-values are shown by the bar plots in the figure.

**SFigure 10. Enrichments of top 50 yeast protein binding motifs at predicted type I and type II protein binding sites.**

**
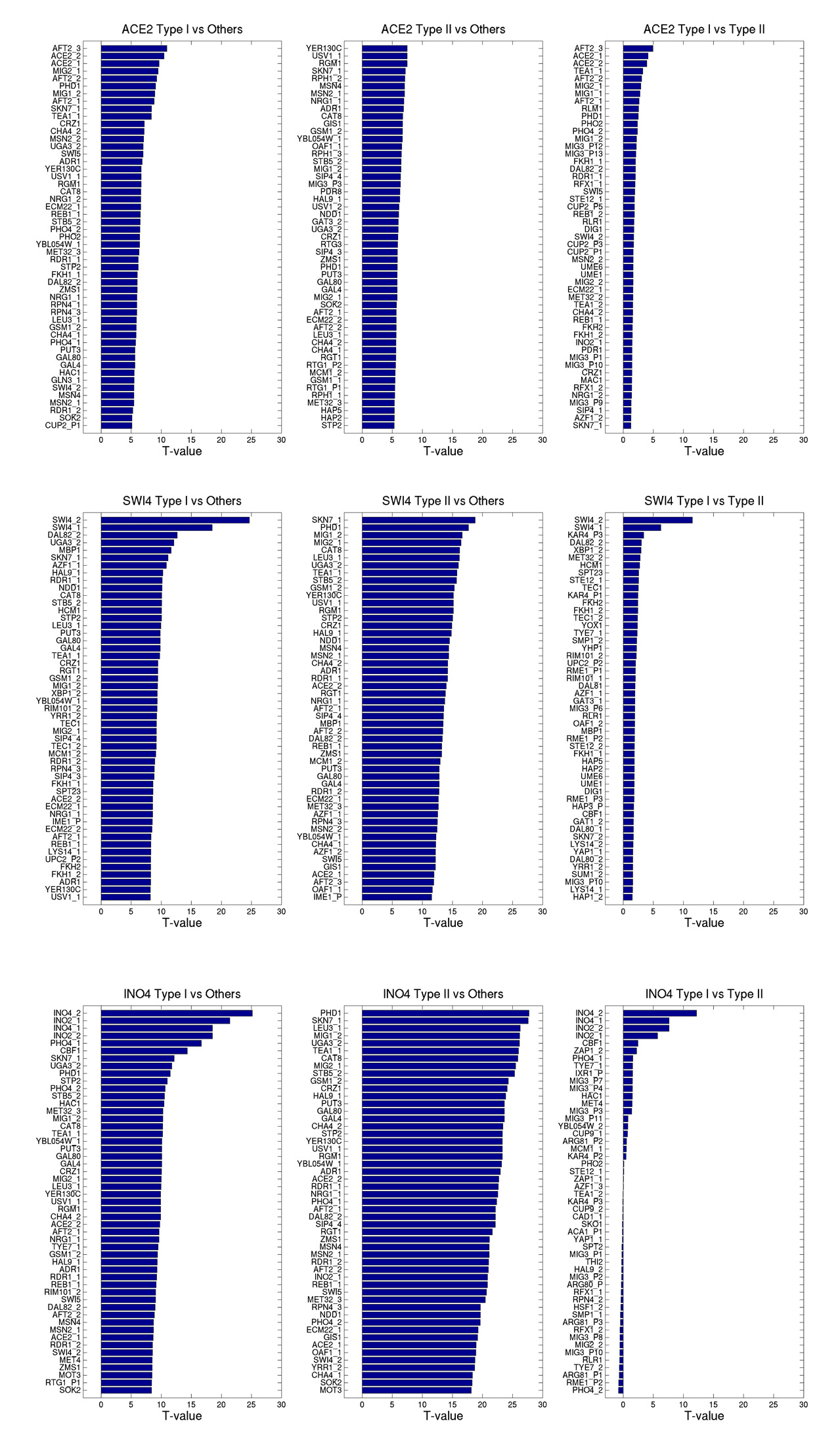
**

In the figure, bar plots of T-values of the top 50 enriched yeast TFs in SFigure 9 are illustrated. Detailed description please refers to SFigure 9.

**SFigure 11. Distribution of ChIP-Seq tag densities in three types of putative ESR1 target genes.**

**
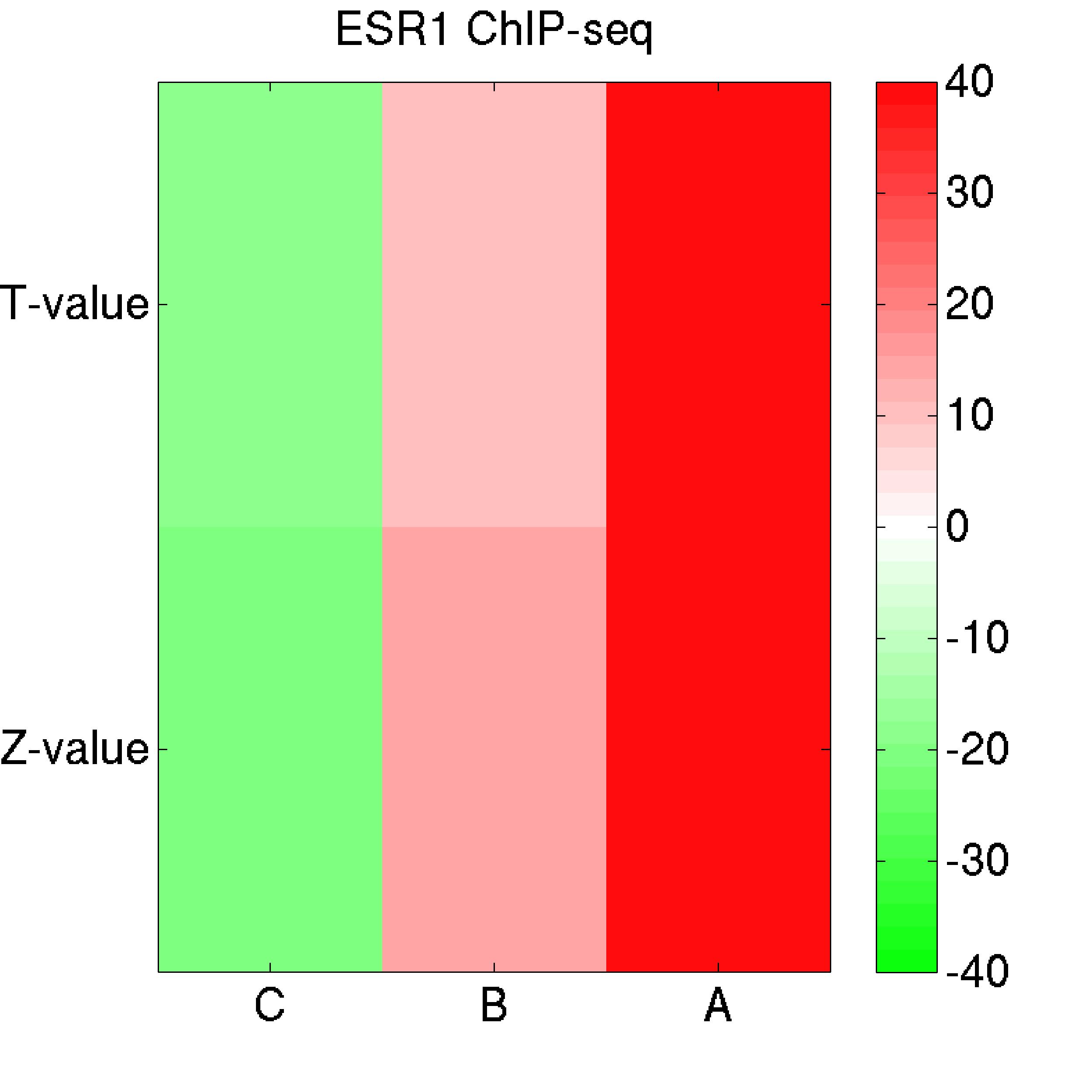
**

In the figure, T-value and Z-value are results of t-test and Mann-Whitney U test, respectively, for ChIP-seq tag density between putative ESR1 target genes (i.e. ‘C’, ‘B’, and ‘A’ genes) and the rest of genes in E2 treated MCF-7 breast cancer cell lines. Positive and negative T-values (Z-values) are colored by red and green, respectively.

**SFigure 12 Differential gene expression activities in three types of putative ESR1 target genes after filtering low binding affinity sites.**

**
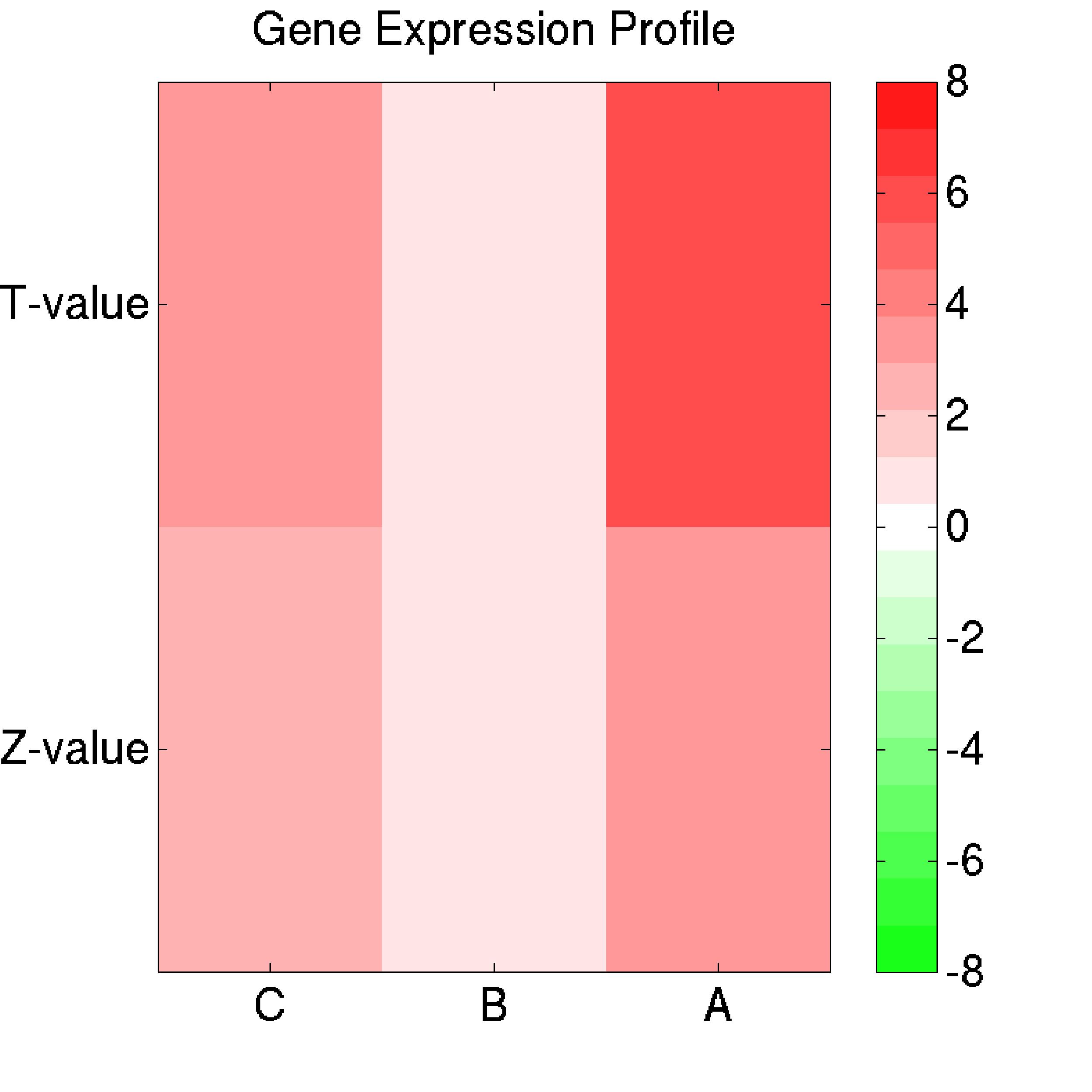
**

In the figure, T-value and Z-value are results of t-test and Mann-Whitney U test, respectively, for gene expression profiles between putative ESR1 target genes (i.e. ‘C’, ‘B’, and ‘A’ genes) and the rest of genes in E2 treated MCF-7 breast cancer cell lines. Positive and negative T-values (Z-values) are colored by red and green, respectively. Here, low binding affinity sites were removed.

**Sfigure 13. Distribution of ChIP-Seq tag densities in three types of putative ESR1 target genes after filtering low binding affinity sites.**

**
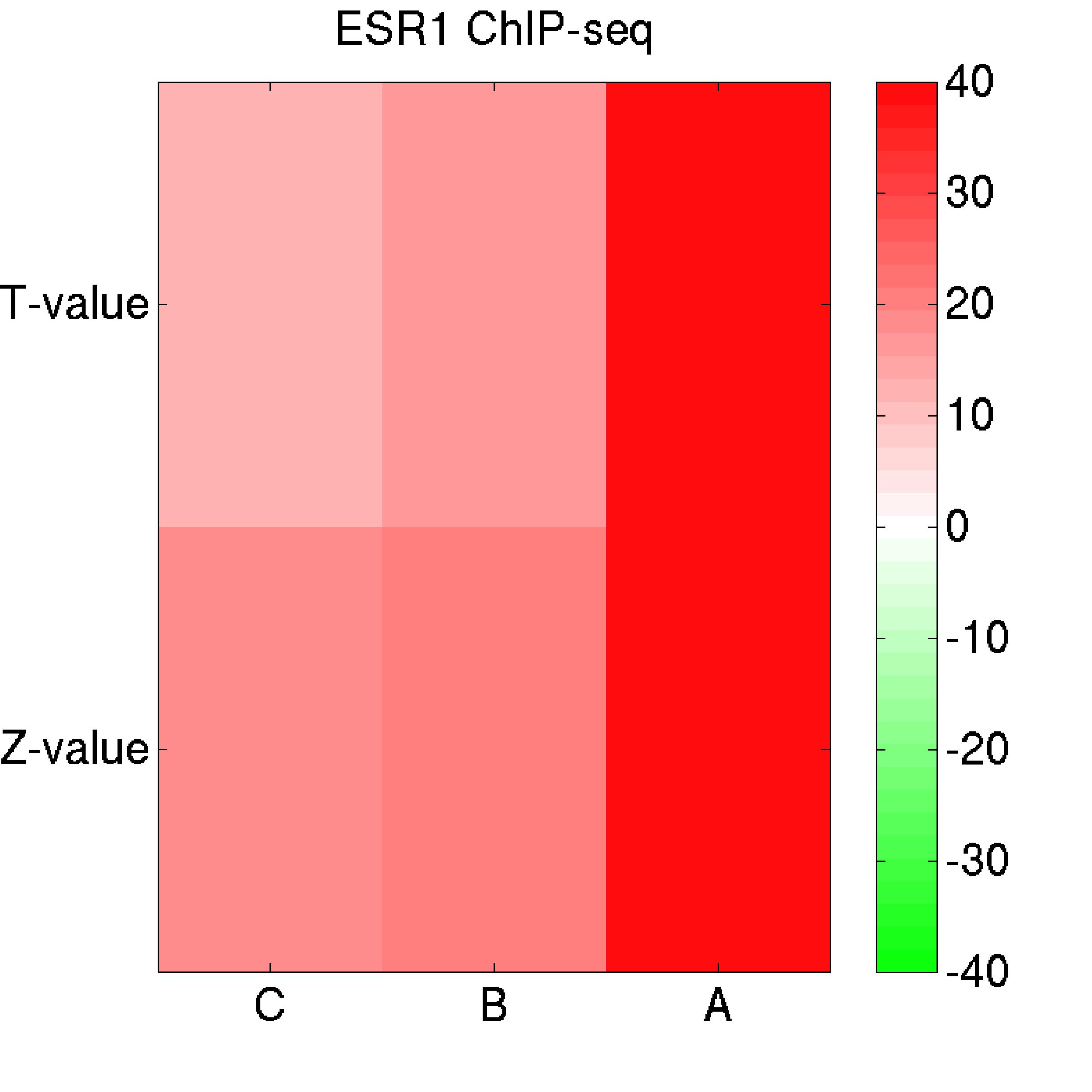
**

In the figure, T-value and Z-value are results of t-test and Mann-Whitney U test, respectively, for ChIP-seq tag density between putative ESR1 target genes (i.e. ‘C’, ‘B’, and ‘A’ genes) and the rest of genes in E2 treated MCF-7 breast cancer cell lines. Positive and negative T-values (Z-values) are colored by red and green, respectively. Here, low binding affinity sites were filtered.

**Sfigure 14 Differential histone modification patterns in three types of putative ESR1 target genes after filtering low binding affinity sites**

**
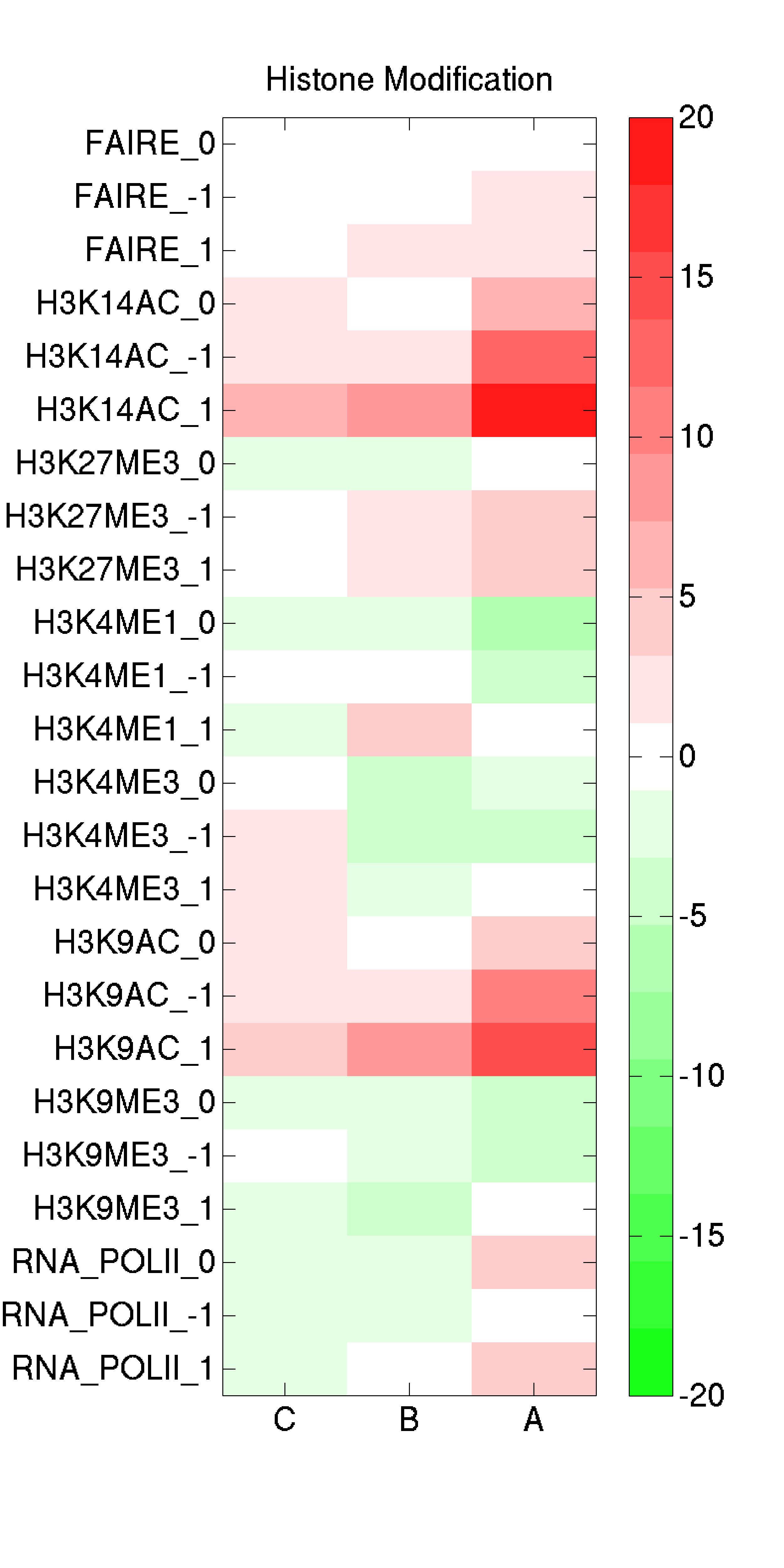
**

Here, T-test was used to evaluate significance of various marks between ESR1 target gene (‘C’, ‘B’, and ‘A’ genes) and the rest of genes in genome. Positive and negative T-values are colored by red and green, respectively. In the figure, 0, +1 and −1 represent histone modifications in E2 treated MCF-7 breast cancer cell lines at gene body, 5 kb upstream and 5 kb downstream, respectively.

**Sfigure 15. Differential gene expression activities in three types of putative SPIB target genes after filtering low binding affinity sites.**

**
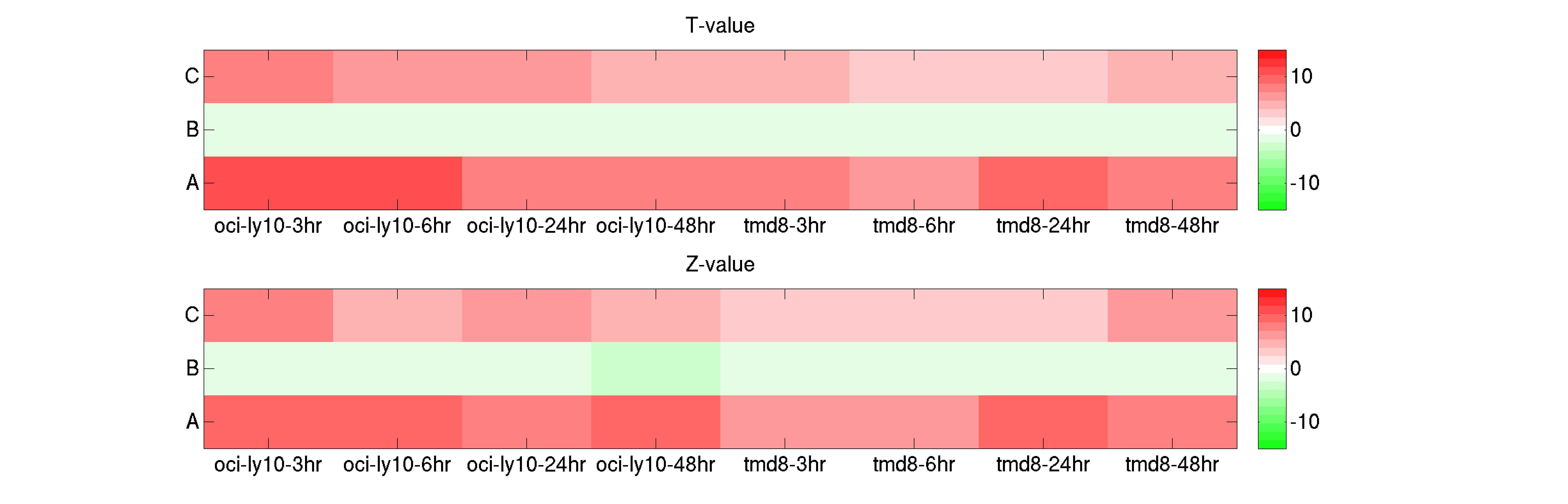
**

In the figure, T-value and Z-value are results of t-test and Mann-Whitney U test, respectively, for gene expression profiles between putative SPIB target genes (i.e. ‘C’, ‘B’, and ‘A’ genes) and the rest of genes in lenalidomide treated ABC DLBCL cell lines (i.e. Oci-ly 10, and TMD8; hr represents treated hours). Positive and negative T-values (Z-values) are colored by red and green, respectively. Here, low binding affinity sites were filtered.

**SFigure 16. Differential gene responses at three types of putative SPIB target genes (low binding affinity sites were filtered) after silencing of SPIB by RNA interference in ABC DLBCL cell line.**

**
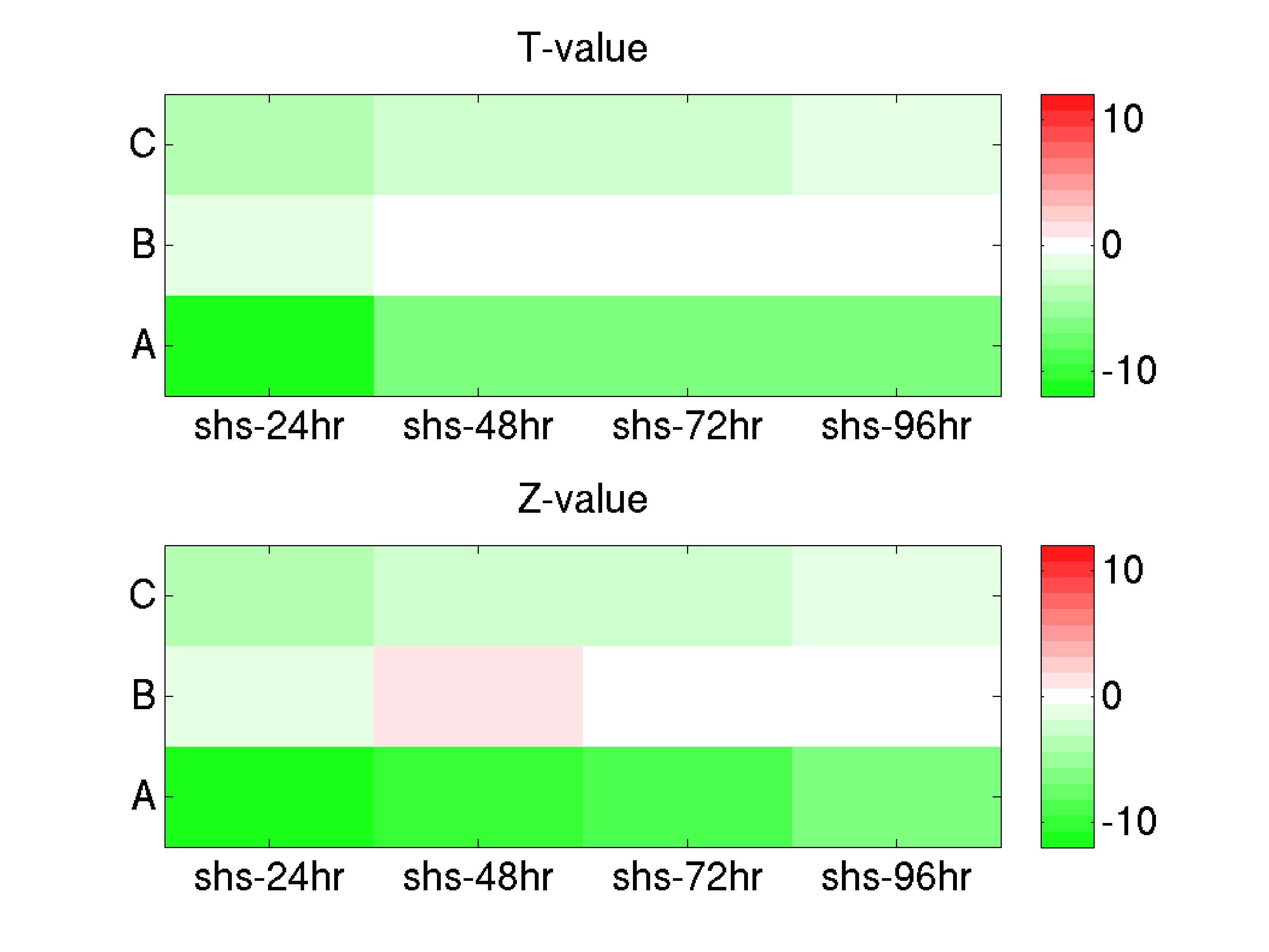
**

In the figure, T-value and Z-value are results of t-test and Mann-Whitney U test, respectively, for gene expression profiles between putative SPIB target genes (i.e. ‘C’, ‘B’, and ‘A’ genes) and the rest of genes after silencing of SPIB by RNA interference in ABC DLBCL cell lines (i.e. HBL1; shs-hr represents silencing of SPIB in hours). Positive and negative T-values (Z-values) are colored by red and green, respectively. Here, low binding affinity sites were filtered.

**SFigure 17. Sequence log representation of predicted PBEMs from type I ESR1 binding sites.
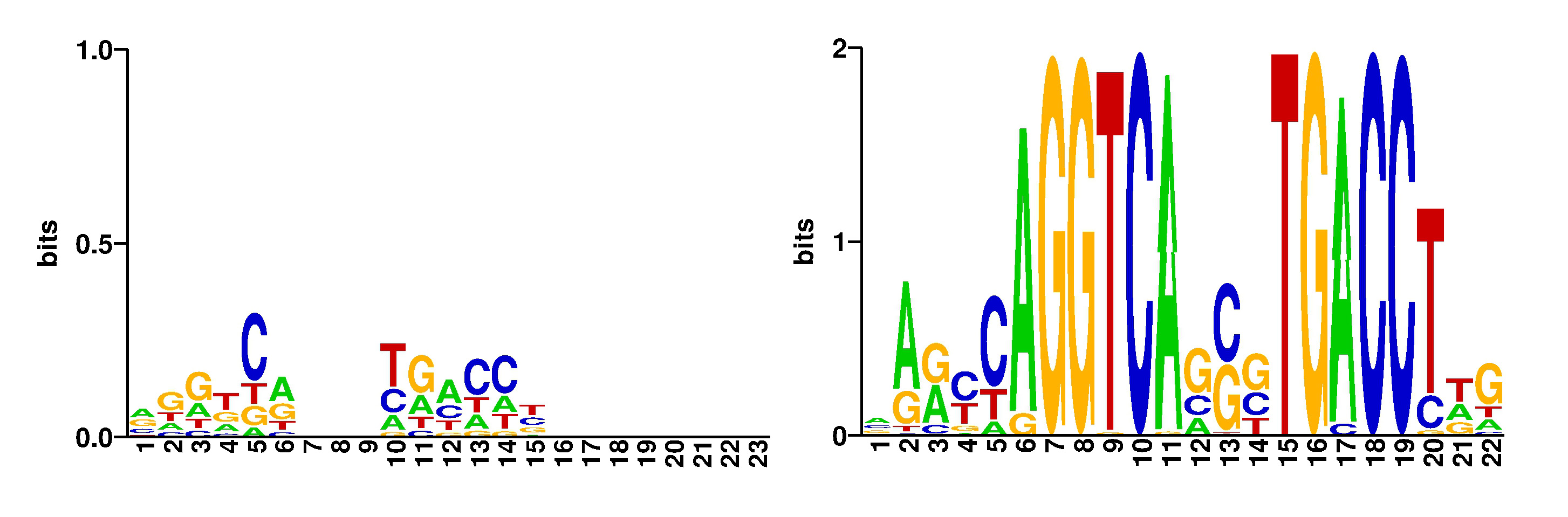
**

Left panel and right panel are the best representative PBEM and the meta-PBEM of ESR1 predicted by applying BayesPI2+ serial computation (100% called peaks) and BayesPI2+ ensemble learning (5% called peaks) on the predicted type I ESR1 binding sites, respectively.

**SFigure 18 Motif enrichment test in predicted type I and type II binding sites.**

**
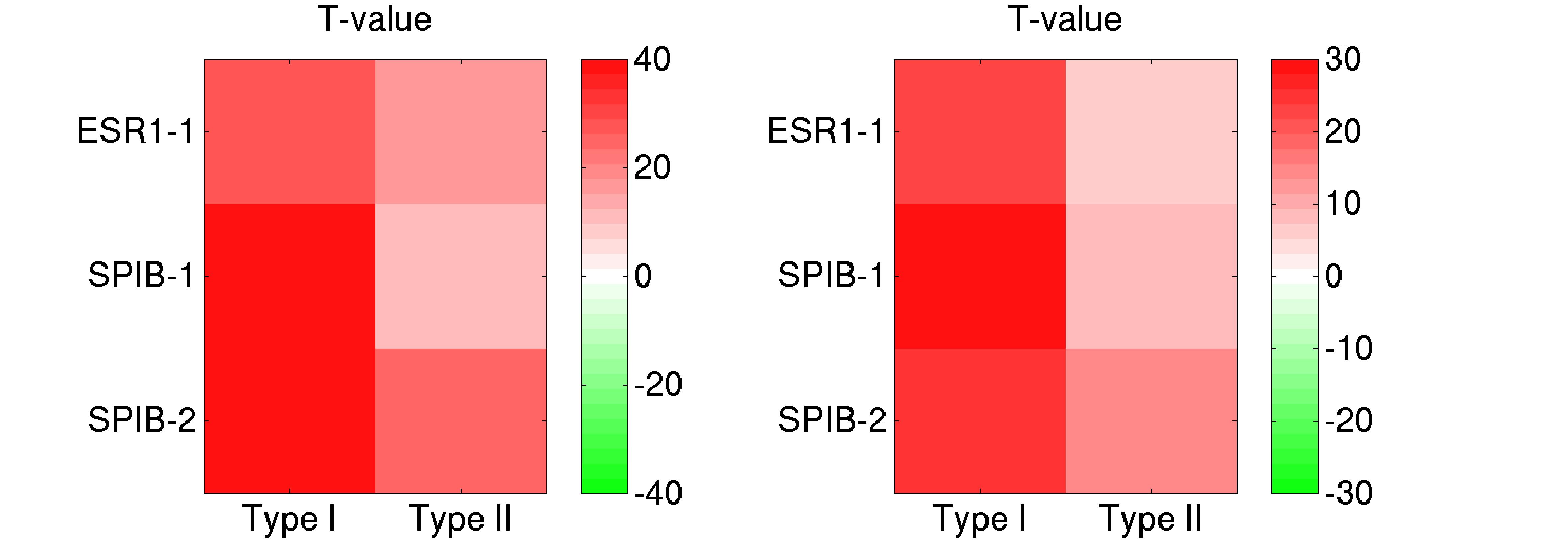
**

In the figure, “type I” and “type II” means predicted type I (direct) and type II (indirect/alternative) protein binding sites, respectively; “ESR1-1” and “SPIB-1” represent ESR1 and SPIB PBEM (motif similar score>0.7) predicted from ESR1 and SPIB type I binding sites, respectively; “SPIB-2” means a SPIB similar PBEM inferred from type II SPIB binding sites. Left and right panels are enrichment tests for predicted PBEMs (i.e. by a serial computation of BayesPI2+) and meta-PBEMs (i.e. by a parallel ensemble learning of BayesPI2+) at type I and type II protein binding sites, respectively. The enrichment is evaluated by fitting the predicted protein binding affinities and the measured ChIP-seq tag densities to a linear regression model, then transforming the regression coefficient to T-value for accessing the significance of predicted PBEMs in either type I or type II protein binding sites.

**SFigure 19. Sequence log representation of predicted meta-PBEMs for primary and alternative SPIB binding.**

**
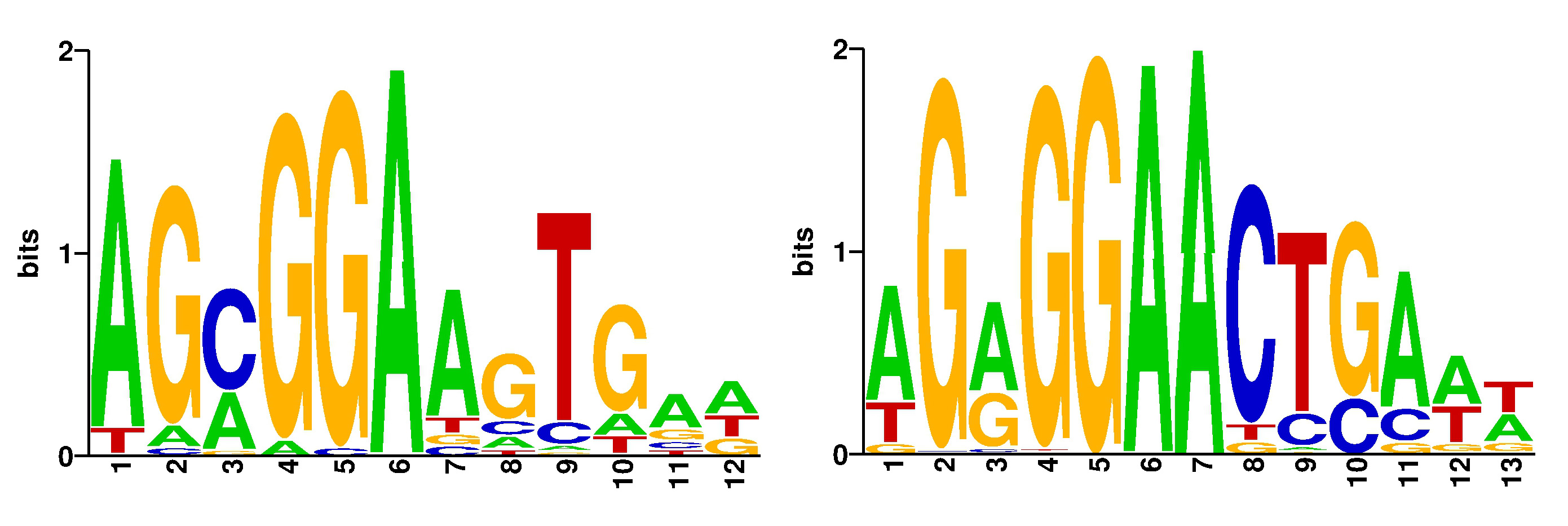
**

Left panel and right panel are predicted primary (inferred from type I SPIB binding sites) and alternative (inferred from type II SPIB binding sits) SPIB binding motif, respectively, by using BayesPI2+ parallel ensemble learning approach.

**SFigure 20 Motif enrichment test in predicted type I and type II binding sites after filtering low binding affinity sites.**

**
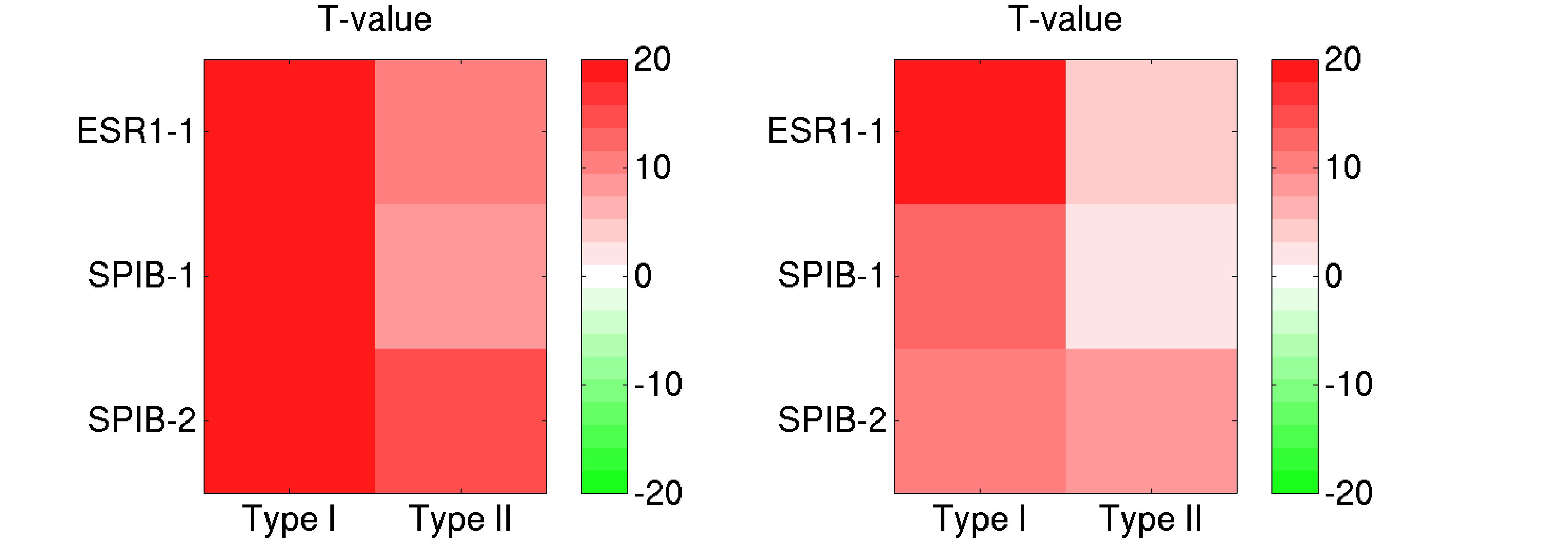
**

In the figure, “type I” and “type II” means predicted type I (direct) and type II (indirect/alternative) protein binding sites, respectively; “ESR1-1” and “SPIB-1” represent ESR1 and SPIB PBEM (motif similar score>0.7) predicted from type I ESR1 and SPIB binding sites, respectively; “SPIB-2” means a SPIB similar PBEM inferred from type II SPIB binding sites. Left panel and right panel are enrichment tests of the predicted PBEMs (i.e. by a serial computation of BayesPI2+) and meta-PBEMs (i.e. by a parallel ensemble learning of BayesPI2+) at type I and type II protein binding sites, respectively. Here, low protein binding affinity sites are removed. The enrichment is evaluated by fitting the predicted protein binding affinity and the measured ChIP-seq tag density to a linear regression model, then transforming the regression coefficient to T-value for accessing the significance of predicted PBEM in either type I or type II protein binding sites.

**SFigure 21. EMSA analysis of both type I SPIB binding probes and mutated type I SPIB binding probes with purified SPIB protein.**

**
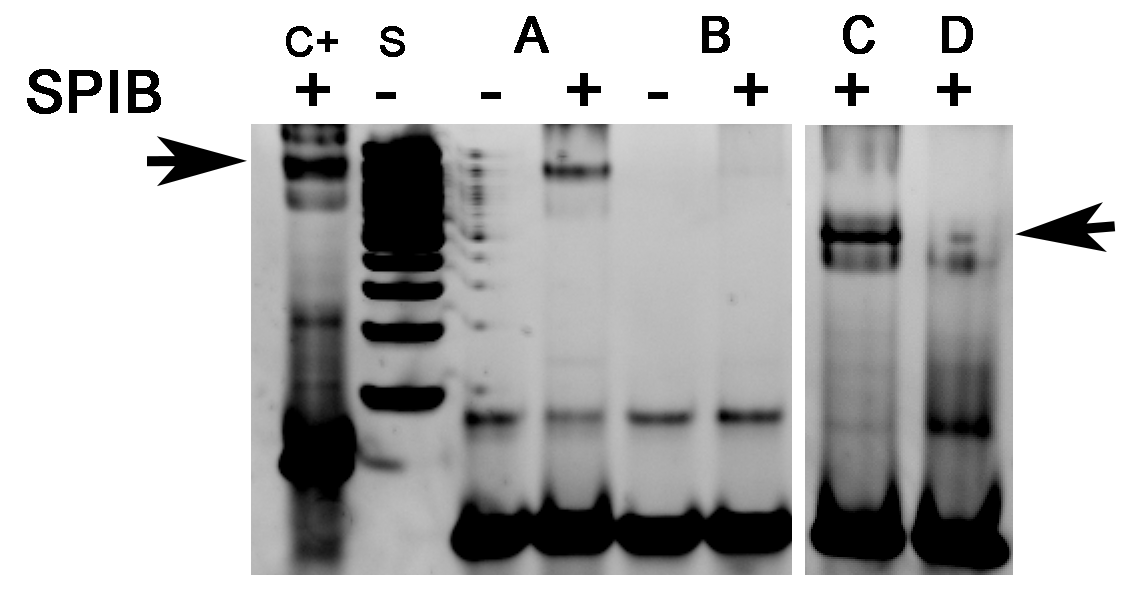
**

SPIB means purified SPIB protein, C+ represents positive control of previously published SPIB binding motif [4] and – is the negative control, S is DNA size standard (lowest band 100bp, highest band 3000bp). The rest of column labels: A and C represent original type I SPIB binding probes in STable 6, and B and D are the corresponding mutated type I SPIB binding probes in STable 6. The black arrow is pointing to the shift band (separately for each gel)

**Supplementary Tables**

Stable 1a Functional annotation of three types of putative ESR1 target genes by using DAVID

|  | Tissue expression | KEGG pathway |
| --- | --- | --- |
| ESR1 ’C’ genes (1130) | prostate_carcinoma_3rd (92 genes)  prostate_adenocarcinoma_3rd (113 genes)  thyroid_3rd (89 genes)  white blood cells_monocyte_3rd (114 genes)  prostate_carcinoma, prostate glands and stroma_3rd (85 genes)  white blood cells_plaque macrophage_3rd (66 genes)  white blood cells_invasive breast cancer, ER+, PR+, Her2-_3rd (70 genes)  mammary gland_breast carcinoma cell line_3rd (126 genes) | Histidine metabolism (6 genes)  Systemic lupus erythematosus (12 genes)  Arginine and proline metabolism (8 genes)  Lysosome (13 genes)  Steroid hormone biosynthesis (7 genes)  Primary immunodeficiency (6 genes)  Metabolism of xenobiotics by cytochrome P450 (8 genes)  Drug metabolism (8 genes)  Oxidative phosphorylation (13 genes) |
| ESR1 ’B’ genes (3636) | brain_anaplastic astrocytoma_3rd (274 genes)  spinal cord_normal spinal cord_3rd (391 genes)  brain_null_3rd (371 genes)  brain_normal thalamus_3rd (378 genes)  brain_Anaplastic GradeIII Astrocytoma_3rd (287 genes)  stem cell_null_3rd (261 genes)  brain_anaplastic astrocytoma_3rd (351 genes)  stomach_gastric cancer_3rd (361 genes)  cerebellum_medulloblastoma, cerebellum_3rd (333 genes) | Melanogenesis (35 genes)  Prostate cancer (32 genes)  Gap junction (31 genes)  Ubiquitin mediated proteolysis (43 genes)  Alanine, aspartate and glutamate metabolism (14 genes)  Endometrial cancer (20 genes)  Non-small cell lung cancer (20 genes)  Vascular smooth muscle contraction (35 genes)  Progesterone-mediated oocyte maturation (28 genes)  Calcium signaling pathway (50 genes) |
| ESR1 ’A’ genes (3180) | mammary gland_invasive breast cancer ER+, PR+, Her2-, grade II_3rd (270 genes)  mammary gland_Grade I, ER+, PR+, Her2- invasive ductal_3rd (245 genes)  mammary gland_Grade I, ER+, PR+, Her2- invasive ductal carcinoma_3rd (180 genes)  mammary gland_ER+, PR+, HER2-, grade II_3rd (281 genes)  mammary gland_ductal carcinoma in situ, extensive, gradeIII, Her2+_3rd (180 genes)  stomach_Stomach, T4N1M0, poorly differentiated carcinoma, obtained by surgery_3rd (228 genes)  mammary gland_ductal carcinoma in situ, extensive, gradeIII, Her2+_3rd (269 genes)  mammary gland_extensive LCIS_3rd (152 genes) | Pathways in cancer (98 genes)  MAPK signaling pathway (77 genes)  Focal adhesion (61 genes)  Adipocytokine signaling pathway (27 genes)  Type II diabetes mellitus (20 genes)  Insulin signaling pathway (40 genes)  Regulation of actin cytoskeleton (57 genes)  Chronic myeloid leukemia (25 genes)  ECM-receptor interaction (27 genes)  Endocytosis (49 genes) |

In the table, top 10 of each functional annotation are presented here, where only one result is shown if multiple annotations with the same name.

**Stable 1b Functional annotation of three types of putative ESR1 target genes (after filtering low binding affinity sites) by using DAVID**

|  | Tissue expression | KEGG pathway |
| --- | --- | --- |
| ESR1 ’C’ genes (922) | white blood cells_invasive breast cancer, ER+, PR+, Her2-_3rd 60  brain_ependymoma_3rd 86  liver_normal bulk liver_3rd 67  brain_Glioblastoma_3rd 77  mammary gland_juvenile fibroadenoma_3rd 93  lung_normal_3rd 82  thyroid__3rd 72  brain_normal thalamus_3rd 101  stomach_null_3rd 72 | Lysosome 14  Arginine and proline metabolism 8  Adipocytokine signaling pathway 8  Steroid hormone biosynthesis 6  Type II diabetes mellitus 6 |
| ESR1 ’B’ genes (3552) | spinal cord_normal spinal cord_3rd 390  brain_null_3rd 374  brain_normal thalamus_3rd 378  brain_astrocytoma grade II_3rd 268  brain_anaplastic gradeIII, primary, brain_3rd 293  peritoneum_normal_3rd 280  white blood cells_monocyte_3rd 257  brain_anaplastic astrocytoma_3rd 257 | Melanogenesis 36  Gap junction 33  Vascular smooth muscle contraction 39  Regulation of actin cytoskeleton 65  Ubiquitin mediated proteolysis 43  ErbB signaling pathway 30  Calcium signaling pathway 52  Purine metabolism 46  Pathways in cancer 87  Focal adhesion 56 |
| ESR1 ’A’ genes (2450) | mammary gland_invasive breast cancer ER+, PR+, Her2-, grade II_3rd 211  mammary gland_Grade I, ER+, PR+, Her2- invasive ductal carcinoma_3rd 143  mammary gland_Grade I, ER+, PR+, Her2- invasive ductal_3rd 192  mammary gland_ductal carcinoma in situ, extensive, gradeIII, Her2+_3rd 214  mammary gland_ER+, PR+, HER2-, grade II_3rd 149  cerebellum_medulloblastoma, cerebellum_3rd 258  mammary gland_extensive LCIS_3rd 120  mammary gland_normal breast organoid_3rd 187 | Pathways in cancer 79  Focal adhesion 48  Insulin signaling pathway 33  MAPK signaling pathway 56  Axon guidance 30  Chondroitin sulfate biosynthesis 9  Aldosterone-regulated sodium reabsorption 13  Wnt signaling pathway 33  mTOR signaling pathway 15  Type II diabetes mellitus 14 |

Here, low affinity ESR1 binding sites were removed before ESR1 target gene assignment, and the top 10 of each functional annotation are listed. If multiple annotations with the same name then only one result is shown.

Table 2a. Functional annotation of three types of putative SPIB target genes by using DAVID

|  | Tissue expression | KEGG pathway |
| --- | --- | --- |
| SPIB ‘C’ genes (675) | colon_cell line derived from colorectal carcinoma_3rd (68 genes)  pancreas_adenocarcinoma_3rd (106 genes)  muscle_normal muscle biopsy of vastus lateralis_3rd (52 genes)  colon__3rd (63 genes)  cerebellum_desmoplastic medulloblastoma_3rd (75 genes)  cartilage__3rd (67 genes)  skin_primary malignant melanoma, vertical growth phase_ level 5_3rd (57 genes) | Parkinson's disease (12 genes)  Huntington's disease (14 genes)  Oxidative phosphorylation (10 genes)  Alzheimer's disease (11 genes)  Galactose metabolism (4 genes)  Limonene and pinene degradation (3 genes) |
| SPIB ‘B’ genes (6344) | brain_null_3rd (569 genes)  stem cell_null_3rd (451 genes)  ovary_normal surface epithelium_3rd (502 genes)  mammary gland_ER neagtive breast cancer cell line_3rd (626 genes)  mammary gland_breast carcinoma cell line_3rd (570 genes)  brain__3rd (534 genes) | Ribosome (42 genes)  Complement and coagulation cascades (34 genes)  Neuroactive ligand-receptor interaction (105 genes)  Base excision repair (19 genes)  Prion diseases (19 genes)  Regulation of actin cytoskeleton (85 genes)  RNA degradation (26 genes)  Proteasome (22 genes)  Biosynthesis of unsaturated fatty acids (12 genes)  Glutathione metabolism (23 genes) |
| SPIB ‘A’ genes (6911) | mammary gland_invasive breast cancer ER+, PR+, Her2-, grade II_3rd (521 genes)  mammary gland_ductal carcinoma in situ, extensive, gradeIII, Her2+_3rd (563 genes)  white blood cells_plaque macrophage_3rd (292 genes)  mammary gland_Grade I, ER+, PR+, Her2- invasive ductal_3rd (479 genes)  mammary gland_normal breast tissue from a breast cancer patient (corresponding to IDC7)_3rd (496 genes)  white blood cells_monocyte_3rd (527 genes)  mammary gland_recurrent phyllodes tumor, malignant, high grade, poorly differentiated_3rd (509 genes) | MAPK signaling pathway (145 genes)  Pathways in cancer (173 genes)  T cell receptor signaling pathway (67 genes)  B cell receptor signaling pathway (49 genes)  Neurotrophin signaling pathway (73 genes)  Fc epsilon RI signaling pathway (49 genes)  Axon guidance (74 genes)  Vascular smooth muscle contraction (65 genes)  Melanogenesis (58 genes)  Fc gamma R-mediated phagocytosis (56 genes) |

In the table, SPIB ‘C’ genes represents putative SPIB target genes regulated by type II SPIB binding sites only, SPIB ‘B’ genes means putative SPIB target genes controlled by type I SPIB binding sites only, and SPIB ‘A’ genes represents putative SPIB target genes regulated by both type I and type II SPIB binding sites. The top 10 of each functional annotation are presented here, where only one result is shown if multiple annotations with the same name.

**Stable 2b Functional annotation of three types of putative SPIB target genes (after filtering low binding affinity sites) by using DAVID**

|  | Tissue expression | KEGG pathway |
| --- | --- | --- |
| SPIB ’C’ genes (1453) | ovary_carcinoma_3rd 234  prostate_carcinoma_3rd 191  colon_cell line derived from colorectal carcinoma_3rd 120  bone__3rd 144  colon__3rd 136  prostate_carcinoma, prostate glands and stroma_3rd 119 | Nucleotide excision repair 8  Complement and coagulation cascades 10  Citrate cycle (TCA cycle) 6  Lysosome 14 |
| SPIB ’B’ genes (4742) | brain_null_3rd 442  stem cell_null_3rd 295  brain_GBM_3rd 371  ovary_normal surface epithelium_3rd 380  uncharacterized tissue_mixture of human cancer cell lines_3rd 449  cartilage_Dedifferentiated chondrosarcoma lung metastasis_3rd 332  prostate_prostate carcinoma_3rd 555  colon__3rd 356  liver_normal bulk liver_3rd 294 | RNA degradation 27  alpha-Linolenic acid metabolism 12  Ribosome 35  Neuroactive ligand-receptor interaction 86  Arrhythmogenic right ventricular cardiomyopathy (ARVC) 31  Folate biosynthesis 8  ABC transporters 19  Complement and coagulation cascades 26  Alanine, aspartate and glutamate metabolism 13  Biosynthesis of unsaturated fatty acids 10 |
| SPIB ’A’ genes (6740) | mammary gland_invasive breast cancer ER+, PR+, Her2-, grade II_3rd 532  mammary gland_ductal carcinoma in situ, extensive, gradeIII, Her2+_3rd 567  mammary gland_normal breast organoid_3rd 499  mammary gland_Grade I, ER+, PR+, Her2- invasive ductal_3rd 483  white blood cells_plaque macrophage_3rd 291  mammary gland_normal breast tissue from a breast cancer patient (corresponding to IDC7)_3rd 323  mammary gland_Grade I, ER+, PR+, Her2- invasive ductal carcinoma_3rd 345  white blood cells_monocyte_3rd 565 | T cell receptor signaling pathway 73  Pathways in cancer 179  B cell receptor signaling pathway 51  MAPK signaling pathway 143  Neurotrophin signaling pathway 74  Wnt signaling pathway 86  Focal adhesion 109  Axon guidance 74  Renal cell carcinoma 44  Jak-STAT signaling pathway 84 |

In the table, low affinity SPIB binding sites were removed before SPIB target gene assignment, and the top 10 of each functional annotation are listed. If multiple annotations with the same name then only one result is shown.

**Stable 3 Functional annotation of the 1687 strongest SPIB ‘A’ genes by using DAVID**

|  | Tissue expression | KEGG pathway | GO |
| --- | --- | --- | --- |
| 1687 strongest SPIB ’A’ target genes | white blood cells_monocyte_3rd 267  lymph node_Large B cell Lymphoma, Malignant, high grade, involving the serosa of the appendix_3rd 224 | B cell receptor signaling pathway 24  Lysosome 28  Neurotrophin signaling pathway 28  Homologous recombination 10  Adipocytokine signaling pathway 16  Chemokine signaling pathway 34  Jak-STAT signaling pathway 29  T cell receptor signaling pathway 22  Cell cycle 24  Pathways in cancer 52 | membrane-enclosed lumen 268  intracellular organelle lumen 258  nuclear lumen 220  organelle lumen 260  leukocyte activation 64  cytosol 199  cell activation 68  lymphocyte activation 52  nucleoplasm 140  establishment of protein localization 132 |

In the table, the 1687 strongest SPIB ‘A’ genes were selected by a two tailed t-test of gene expression profiles between lenalidomide treated ABC DLBCL cell lines and silencing of SPIB in ABC DLBCL cell lines. Here, the top 10 of each functional annotation are presented. If multiple annotations with the same name then only one result is shown.

**STable 4. Selected 10 type I SPIB binding sites from CD-anti-genes that are included in the 1687 strongest SPIB ‘A’ genes**

| ID | Probe name (chromosome location) | Sequence | ChIP-Seq Tag |
| --- | --- | --- | --- |
| 1 | CXCR4 chr2:136880451_(-4726) | gtaatttattggtggtgacctcagacagctatataaaaagaggaagcgcc | 36 |
| 2 | IGF2R chr6:160380371_(-9760) | atttcctctttttcagaggatggcatcaccaaatagtcatggtgccagaa | 115 |
| 3 | CD72 chr9:35619591_(-1167) | gagctctgccaatagctccgcccagccagccctagaggaagtggccactg | 137 |
| 4 | CD44 chr11:35087731_(-72686) | ataccataggcttatgacttctcagttcctcttaataggattcaagccac | 41 |
| 5 | CD44 chr11:35128371_(-32046) | atcaacgagatgttttcacttctggtttctgagaggaactggcaggatat | 144 |
| 6 | CCR7 chr17:38729021_(-7285) | tcagggaagagaaaggaataaagtgtactcaaataggaagagaggaagtg | 34 |
| 7 | CCR7 chr17:38771651_(-49915) | ccttcccagggttaagagagagaagaggaagtgaaaacaggaggggagaa | 112 |
| 8 | CD47 chr3:107843351_(-33416) | agaggaagtgaatgaaagctcttctttctcctctgctgctttctttgtcc | 101 |
| 9 | CD74 chr5:149811361_(-18862) | tttgaagccaggtctgagctgtctccagagcacttcctctcaatccccat | 43 |
| 10 | IL10RA chr11:117881871_(+24765) | agggccctaaaagaggaagtcgcccagtatcagttctgctcccgaggatg | 328 |

In the table, ID is the probe ID in Figure 10; Probe name is annotated SPIB target gene to the binding site, where + and – values are distance to TSS from downstream and upstream, respectively; sequence is 50bp probe sequence that is used in EMSA analysis; ChIIP-seq Tag indicates the measured raw tag counts from SPIB ChIP-seq experiment in ABC DLBCL cell lines. Here, probe that did not observe shifted band in Figure 10 is marked by yellow color.

**Stable 5. Selected 10 type II SPIB binding sites from CD-anti-genes that are included in the 1687 strongest SPIB ‘A’ genes**

| ID | Probe name (chromosom location) | Sequence | ChIP-seq Tag |
| --- | --- | --- | --- |
| 11 | CD82 chr11:44631001_(+43860) | tgctgtcacgctcaagcagaagacattggaggggaaggggctcacttggc | 57 |
| 12 | LAG3 chr12:6880471_(-1199) | agaaacagcccagaaacaggaagtccttcccctgagctgggagagggctg | 133 |
| 13 | CD300LD chr17:72603691_(-15321) | ctgaatgaggttgaagattggccaagagtagtgtcaggagccatggaggc | 39 |
| 14 | CD1D chr1:158085111_(-64626) | gatgactgagctgtgggcccttggttacttacctcatctctcgagtctca | 48 |
| 15 | CD1D chr1:158120451_(-29286) | ggacctaaccctgggttgacttctaggtcagagagtgggaagtgaaacaa | 46 |
| 16 | CD47 chr3:107819901_(-9966) | Tctggagaatgaaggaaatgaaactgggggatcccctagaggaacgacag | 77 |
| 17 | IGF2R chr6:160382731_(-7400) | ggtttcctctttttgaagctgggtagggcttggtgactgtcctgatcaca | 37 |
| 18 | CD274 chr9:5451691_(+1188) | tcaaggaagtcacagaaatactgtggggaattgaaaccccatgtggaaaa | 30 |
| 19 | CXCR3 chrX:70849521_(-11154) | Gatgaggagacccaggggaggaagcaggaagtttcagtaccctatgactg | 70 |
| 20 | CXCR4 chr2:136890081_(-14356) | agcgacagagagttatgacctcgaacccacaagtgagactgcaaacacaa | 50 |

In the table, ID is the probe ID in Figure 10; Probe name is annotated SPIB target gene to the binding site, where + and – values are distance to TSS from downstream and upstream, respectively; sequence is 50bp probe sequence that is used in EMSA analysis; ChIIP-seq Tag indicates the measured raw tag counts by SPIB ChIP-seq experiment in ABC DLBCL cell lines. Here, probes that did not observe shifted band in Figure 10 are marked by yellow color.

**Stable 6. Selected 2 type I SPIB binding sites for SPIB binding specificity test**

| ID | Probe name (chromosom location) | Sequence | ChIP-seq Tag |
| --- | --- | --- | --- |
| A | CD44 chr11:35111511_(-48906) | Tgaaaaatccctctcccactt**cc**tcttctg | 140 |
| B | CD44 chr11:35111511_(-48906)- mutated | Tgaaaaatccctctcccactt**aa**tcttctg |  |
| C | CD44 chr11:35136591_(-23826) | tagtgaggagcagagctaggattcagtt**cc**tcttt**cc**atctcctctgtga | 226 |
| D | CD44 chr11:35136591_(-23826)-mutated | tagtgaggagcagagctaggattcagtt**aa**tcttt**aa**atctcctctgtga |  |

In the table, ID is probe ID in Supplementary Figure 21 (i.e. ID (A, C) are selected type I SPIB binding sites, and ID (B, D) are corresponding mutated probe), the mutated 2bp are marked by underline in the sequence, ChIP-seq Tag indicates the measured raw tag counts at the binding sites by SPIB ChIP-seq experiment in ABC DLBCL cell lines.

**Supplementary References**

**1. Wang J, Bo TH *et al*: Tumor classification and marker gene prediction by feature selection and fuzzy c-means clustering using microarray data. *BMC bioinformatics* 2003, 4:60.**

**2. Teixeira MC, Monteiro P *et al*: The YEASTRACT database: a tool for the analysis of transcription regulatory associations in Saccharomyces cerevisiae. *Nucleic acids research* 2006, 34(Database issue):D446-451.**

**3. Harbison CT, Gordon DB *et al*: Transcriptional regulatory code of a eukaryotic genome. *Nature* 2004, 431(7004):99-104.**

**4. Dekoninck A, Calomme C *et al*: Identification and characterization of a PU.1/Spi-B binding site in the bovine leukemia virus long terminal repeat. *Oncogene* 2003, 22(19):2882-2896.**
